# Changes in oak (*Quercus robur*) photosynthesis after winter moth (*Operophtera brumata*) herbivory are not explained by changes in chemical or structural leaf traits

**DOI:** 10.1371/journal.pone.0228157

**Published:** 2020-01-24

**Authors:** Kristiina Visakorpi, Terhi Riutta, Yadvinder Malhi, Juha-Pekka Salminen, Norma Salinas, Sofia Gripenberg

**Affiliations:** 1 Department of Zoology, University of Oxford, Oxford, England, United Kingdom; 2 Environmental Change Institute, School of Geography and the Environment, University of Oxford, Oxford, England, United Kingdom; 3 Department of Life Sciences, Silwood Park Campus, Imperial College London, Ascot, England, United Kingdom; 4 Natural Chemistry Research Group, Department of Chemistry, University of Turku, FI Turku, Finland; 5 Seccion Química, Pontificia Universidad Católica del Peru, Lima, Peru; 6 School of Biological Sciences, University of Reading, Reading, England, United Kingdom; Umeå Plant Science Centre, Umeå University, SWEDEN

## Abstract

Insect herbivores have the potential to change both physical and chemical traits of their host plant. Although the impacts of herbivores on their hosts have been widely studied, experiments assessing changes in multiple leaf traits or functions simultaneously are still rare. We experimentally tested whether herbivory by winter moth (*Operophtera brumata*) caterpillars and mechanical leaf wounding changed leaf mass per area, leaf area, leaf carbon and nitrogen content, and the concentrations of 27 polyphenol compounds on oak (*Quercus robur*) leaves. To investigate how potential changes in the studied traits affect leaf functioning, we related the traits to the rates of leaf photosynthesis and respiration. Overall, we did not detect any clear effects of herbivory or mechanical leaf damage on the chemical or physical leaf traits, despite clear effect of herbivory on photosynthesis. Rather, the trait variation was primarily driven by variation between individual trees. Only leaf nitrogen content and a subset of the studied polyphenol compounds correlated with photosynthesis and leaf respiration. Our results suggest that in our study system, abiotic conditions related to the growth location, variation between tree individuals, and seasonal trends in plant physiology are more important than herbivory in determining the distribution and composition of leaf chemical and structural traits.

## Introduction

Insect herbivores are one of the most abundant groups of organisms on Earth, and folivory is one of the most common feeding strategies used by these insects [1,2]. By feeding on leaf tissue, insect herbivores can change a range of chemical and morphological leaf traits [3,4]. These changes are often induced as defences against the herbivores [3] and may result in further alterations in plant function, for example in photosynthesis [5,6] or growth [7,8]. The herbivory-induced changes in plant function are often thought to arise through trade-offs between growth and defence, due to limitations in resource allocation [9,10] or restrictions in the hormonal signalling network [11]. By altering leaf trait composition, herbivory can have large-scale consequences for ecosystem-level processes by influencing rates of carbon sequestration [12] or litter decomposition [13–15].

Plant responses to herbivory can vary widely depending on, for example, the species identity of the herbivore and the host plant. Typically, herbivory results in decreased leaf nutrient content and increased concentrations of chemical plant defences [16–18], and often affects several traits concurrently. For example, herbivory can result in higher concentration of defensive compounds and in decreased nitrogen content, making the plant less palatable and less nutritious [9,19]. Higher investment in defence can slow plant growth and result in smaller leaf size [8,20], increase leaf mass per area due to increased carbon deposition into defensive compounds [21] and suppress photosynthesis [5,6]. Since nitrogen is part of the enzymes important for photosynthesis, leaf nitrogen content is often positively correlated with photosynthetic rate [22,23]. Finally, the concentration and effectiveness of many of the defensive compounds often depends on other leaf traits or on the concentration of other phytochemicals [24,25]. Thus, examining changes in the overall composition of leaf traits is important for understanding the effects of herbivory on leaf structure, chemistry and function.

In this study, we investigated how leaf damage affect selected physical and chemical leaf traits, and how the studied traits correlate with previously documented damage-induced changes in leaf photosynthetic rate [12]. As a study system, we used the pedunculate oak (*Quercus robur* L.) and one of its most common insect herbivores, caterpillars of the winter moth (*Operophtera brumata* L.). Among the potential changes in oak foliage triggered by caterpillar feeding are changes in concentration of different polyphenols [26–28], especially hydrolysable tannins and flavonols. Hydrolysable tannins are found in high concentrations in trees defoliated by insects [16,18] or treated with jasmonic acid [29], and have high oxidative activity in caterpillar guts [30]. Flavonols are often induced after herbivore attacks [31,32]. Thus, both groups of compounds could be expected to respond to herbivory, and to potentially trigger resource re-allocation between leaf function and defence. A third group of polyphenols, proanthocyanidins have also been reported to increase after defoliation [16,33,34], but nevertheless, several studies suggest that they might not be important anti-herbivore defences [4,28,32,35–37].

We used a manipulative field experiment to create two types of damage on oak leaves: feeding damage by caterpillars and mechanical wounding. We measured leaf polyphenol chemistry, leaf nitrogen and carbon content and leaf structure (leaf area and leaf mass per area). We used photosynthesis and leaf respiration measurements previously reported from the same experiment [12] to assess the link between the studied chemical and structural traits and leaf functioning. In our previous study, we showed that photosynthetic rate (light-saturated photosynthesis and electron transport rate) was significantly lower both in leaves damaged by herbivores and in intact leaves growing on the same shoots as these damaged leaves, compared with intact leaves surrounded only by other intact leaves. In this study, we use the same experimental set-up to explore whether the observed changes in photosynthesis can be explained by concurrent changes in leaf traits hypothesised to be influenced by herbivory. Because the herbivory-induced changes in photosynthesis were observed at the level of individual leaves, we expect that any herbivory-associated changes in leaf traits will also be detected at the leaf level.

To evaluate the current evidence of the role of the studied polyphenols in plant defence against herbivores, we conducted a literature review on the relationship between herbivory and the individual polyphenol compounds measured in this study (see File C in S1 Appendix). We related the evidence gathered from the literature to two chemical characteristics of the compounds presumed to describe their anti-herbivore activity: oxidative activity and protein precipitation activity [38,39].

Specifically, we ask:

1. Does experimentally applied herbivory and/or mechanical leaf damage change chemical and structural leaf traits? If so, are these changes dependent on the type of damage (insect herbivory or mechanical damage) and can they also be seen in intact leaves on the same shoots?
2. Are previously observed damage-induced changes in photosynthesis linked to parallel changes in structural and chemical leaf traits?
3. Does the published literature provide evidence for individual compounds functioning as anti-herbivore defences? If so, are compounds with higher oxidative or protein precipitation capacity more likely to show evidence for anti-herbivore activity?

Based on the current literature on polyphenols, induced defences, and trade-offs between plant growth and defence, we predicted that 1) herbivory and/or mechanical leaf damage will result in increased investment in polyphenols (reflected as either increased concentration, diversity, or altered composition of polyphenols) [16,18] and 2) photosynthetic rate will correlate negatively with investment in polyphenols [5,6]. Based on literature on leaf nutrients and physical traits, we predicted that 3) leaf mass per area will increase after herbivory [21], 4) leaves subject to herbivory will grow smaller [8,20], and that 5) leaf nitrogen content will correlate positively with photosynthetic rate [23].

## Materials and methods

### Study system and experimental design

Field work permission was granted by the University of Oxford. The field experiment was carried out on ten oak trees in Oxfordshire, UK. Five of the studied oaks were mature trees (mean diameter at breast height, “dbh” 67.2 cm) located in Wytham Woods (51.7743°, -1.3379°), where their foliage could be accessed from an elevated canopy walkway. The other five trees were young (mean dbh 13.6 cm) planted oaks by the John Krebs field station in Wytham (51.7837°, -1.3170°). At both sites, oaks are naturally infested by caterpillars of the winter moth, which is a common generalist early-spring herbivore. The caterpillars emerge in synchrony with budburst and feed on the newly flushed leaves until early June [40]. Relatively few free-feeding herbivore species feed on the mature oak leaves later in the season [26].

The experiment was conducted during the spring and summer of 2015 and 2016. Between 11^th^ and 15^th^ May 2015 and 9^th^ and 11^th^ May 2016, when leaves were still young, we identified 15 shoots with only intact leaves from each of the study trees and enclosed each shoot in a small mesh fabric (mesh size < 1mm) bag. We randomly assigned each bag into one of three treatments: 1) *herbivore addition*, 2) *mechanical damage*, or 3) *control*, so that each tree had five bags of each treatment. For each of the *herbivore addition* bags we added one locally collected winter moth caterpillar, and let it feed on the leaves for 3–5 days, so that the amount of feeding damage on these leaves was similar to natural levels of herbivory in the area [12]. To ensure that the artificially applied mechanical damage would mimic the herbivory treatment, each shoot assigned to the *mechanical damage* treatment was paired with an herbivore addition shoot. The type and amount of damage on the herbivory addition shoots was then replicated on the mechanical damage shoots by tearing leaf edges or by punching holes. *Control* shoots were left intact. To prevent additional herbivory, we left the mesh bags around the shoots until 25^th^ June 2015 or 28^th^ June 2016, when the amount of insect herbivory had levelled off. Altogether, in each study year there were 15 experimental shoots in each of the ten trees (total n = 300 shoots). For further details on the experimental set-up, see [12].

On shoots in the *herbivory addition* and *mechanical damage* treatments, we measured leaf traits for both damaged and intact leaves. Since no leaves were damaged on *control* shoots, we only measured intact leaves from these shoots. This setup allowed us to measure five leaf-level treatments: *damaged leaf in herbivory treatment*, *undamaged leaf in herbivory treatment*, *damaged leaf in mechanical treatment*, *undamaged leaf in mechanical treatment*, and *intact control leaf*.

### Collecting leaf traits

**Fig 1:**
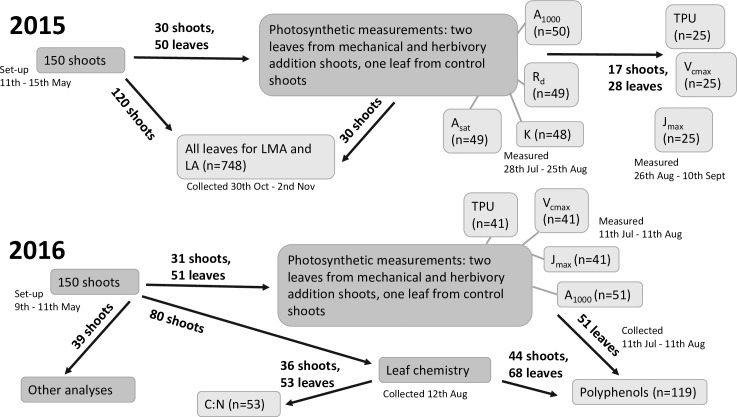
The allocation of experimental leaves to different leaf trait measurements in 2015 and 2016. For abbreviations of leaf traits, see “Collecting leaf traits” above and Table 1. The photosynthetic parameters (A_1000_, A_sat_, K, R_d_, V_cmax_, J_max_, TPU) were derived from photosynthesis-light and photosynthesis-CO_2_ (i.e. A/C_i_) response curves and subsequent model fitting (see [12]). Note that model fitting to calculate some of these parameters failed, hence the final sample size is less than the number of leaves allocated for that trait (41 leaves instead of 51 leaves 2016 for V_cmax_, J_max_, TPU). One outlier of K was removed from further analyses.

Due to time constraints and the limited number of experimental shoots per tree, the data collection of leaf traits spanned two growing seasons, with the timing and number of replicates varying depending on the trait. The data collection stream is summarised in Fig 1. For a detailed description of the methods, see File A in S1 Appendix.

*Chemical traits—*We measured concentrations of polyphenols, leaf nitrogen (N) and leaf carbon (C) on leaves collected in 2016. Polyphenol content was measured on the same leaves that were used for photosynthesis measurements (n = 51 leaves, collected 11^th^ July to 11^th^ August 2016) and from an additional set of leaves obtained from the experiment (n = 68 leaves, collected 12^th^ August 2016). A subset of the remaining leaves were used to analyse carbon and nitrogen content (n = 53 leaves, collected 12^th^ August 2016). Given the current evidence on the role of hydrolysable tannins and flavonols as anti-herbivore defences on oak (see Introduction), we focused our polyphenol analyses on individual compounds within these two groups. Analyses of polyphenol concentrations were carried out in the laboratory of the Natural Chemistry Research Group at the University of Turku, Finland, using ultra-performance liquid chromatography high-resolution mass spectrometry (UPLC-HR-MS). For each sample, we obtained estimates of the concentrations (mg/g) of 27 polyphenol compounds (Table A in S1 Appendix). Analyses of the leaf carbon and nitrogen content were carried out in the Pontificia Universidad Católica del Perú where C and N content (%) was determined by combustion analysis.

*Physical traits*—To calculate leaf mass per area (LMA, g/m^2^) and leaf area (LA, cm^2^), we used leaf material collected at the end of the 2015 field season (103 shoots, 616 leaves; collected 30^th^ October– 2^nd^ November 2015). Leaves were scanned, oven-dried and weighted. The remaining and original leaf area were estimated using ImageJ software (NIH, MD, USA).

*Leaf functioning*–To assess leaf functioning in terms of leaf gas exchange, we used seven photosynthetic parameters obtained from the same experiment during 2015 and 2016 [12]: light-saturated photosynthetic rate at 2000 μmol m^−2^ s^−1^ of photosynthetically active radiation (A_sat_), light intensity where photosynthetic rate is half of its maximum (K), dark respiration rate (R_d_), maximum carboxylation rate (V_cmax_), maximum electron transport rate (J_max_), maximum triose phosphate use efficiency (TPU), and photosynthetic rate in 1000 μmol m^-2^ s^-1^ of photosynthetically active radiation (A_1000_), representing a standard photosynthetic rate in full daylight (Table 1). For summary of all traits measured including sample sizes, see Table 1.

**Table 1:** Summary of the investigated leaf traits, their mean values across all samples and treatments (“Grand mean”) and for each leaf-level treatment separately, and the total number of samples used to obtain values for each trait. The photosynthetic parameters (A_1000_ to TPU) were obtained from an earlier study based on the same experiment [12], the other traits were collected for this study. See Table A in S1 Appendix for concentrations of individual polyphenol compounds. The errors are ± 1 SEM.

Leaf trait	Description	Grand mean	Control	Herbivore damage	Herbivore intact	Mechanical damage	Mechanical intact	n
A_1000_	Photosynthetic rate in 1000 PAR, μmol CO_2_ m^-2^ s^-1^	11.6 ± 0.5	10.8 ± 1.4	8.34 ± 1.2	7.10 ± 0.9	11.6 ± 1.2	9.22 ± 1.1	100
A_sat_	Light-saturated photosynthetic rate, μmol CO_2_ m^-2^ s^-1^	15.3 ± 0.9	19.8 ± 2.2	10.8 ± 1.6	12.5 ± 1.9	17.6 ± 1.7	16.0 ± 1.5	49
K	Light intensity where photosynthetic rate is half of its maximum, μmol m^-2^ s^-1^	168 ± 12	197 ± 29	131 ± 25	130 ± 24	189 ± 21	191 ± 28	48
R_d_	Dark respiration rate, μmol CO_2_ m^-2^ s^-1^	-0.46 ± 0.1	-0.41 ± 0.1	-0.45 ± 0.1	-0.54 ± 0.1	-0.46 ± 0.1	-0.45 ± 0.1	49
V_cmax_	Carboxylation efficiency, μmol CO_2_ m^-2^ s^-1^	70.1 ± 4.3	80 ± 9.3	65.4 ± 9.6	56.9 ± 6.4	84.1 ± 10	67.3 ± 12	65
J_max_	Electron transport efficiency, μmol CO_2_ m^-2^ s^-1^	158.1 ± 11	197 ± 22	147 ± 22	122 ± 16	188 ± 27	146 ± 32	65
TPU	Triose phosphate use efficiency, μmol CO_2_ m^-2^ s^-1^	9.76 ± 0.6	11.7 ± 1.2	8.93 ± 1.1	8.23 ± 0.9	11.4 ± 1.5	8.89 ± 1.7	65
LMA	Leaf mass per area, gm^-2^	63.2 ± 0.6	60.4 ± 1.2	64.4 ± 1.2	66.4 ± 1.5	62.3 ± 1.4	62.5 ± 1.6	616
LA	Estimated original leaf area, cm^2^	18.1 ± 0.5	17.9 ± 1.0	17.7 ± 1.0	18.0 ± 1.3	18.0 ± 1.1	19.3 ± 1.6	616
N	Nitrogen content, %	2.24 ± 0.04	2.34 ± 0.07	2.19 ± 0.08	2.09 ± 0.08	2.30 ± 0.09	2.26 ± 0.10	53
C	Carbon content, %	47.1 ± 0.4	46.7 ± 0.9	46.8 ± 0.9	48.5 ± 1.6	46.8 ± 0.63	47.0 ± 1.0	53
Total phenols	Total concentration of all phenolic compounds, mg/g	34.0 ± 1.1	34.1 ± 2.3	35.4 ± 2.3	32.9 ± 2.4	32.6 ± 2.3	34.9 ± 2.8	119
Phenol diversity	Shannon's diversity index for all phenolic compounds	2.14 ± 0.02	2.14 ± 0.1	2.12 ± 0.04	2.16 ± 0.1	2.16 ± 0.04	2.11 ± 0.04	119
Total HT	Total concentration of hydrolysable tannins, mg/g	24.4 ± 1.2	24.2 ± 2.7	26.1 ± 2.5	22.4 ± 3.0	23.8 ± 2.41	25.1 ± 3.07	119
HT diversity	Shannon's diversity index for hydrolysable tannins	1.56 ± 0.01	1.58 ± 0.04	1.55 ± 0.03	1.59 ± 0.04	1.56 ± 0.03	1.54 ± 0.03	119
Total FL	Total concentration of flavonols, mg/g	5.16 ± 0.2	5.08 ± 0.4	5.26 ± 0.6	5.85 ± 0.5	4.46 ± 0.3	5.32 ± 0.7	119
FL diversity	Shannon's diversity index for flavonols tannins	1.43 ± 0.01	1.44 ± 0.03	1.45 ± 0.02	1.44 ± 0.03	1.44 ± 0.03	1.40 ± 0.03	119

### Statistical analyses

To test if any of the leaf traits differed between the experimental treatments, we used linear mixed effect models and linear multivariate models. To test for relationships between individual chemical or physical leaf traits and photosynthetic or respiration rate, we used mixed effect models, linear models and correlation analyses. To investigate whether the composition of leaf traits was affected by the treatments, we performed Principal Component Analyses (PCA), and Redundancy Analyses (RDA). When combining trait data measured from different leaves and in different years (see Fig 1), we used tree × treatment -specific averages of the measured traits (n = 10 per treatment). When the traits were measured on the same leaf (photosynthesis and polyphenol content), we used leaf-specific values. To account for differences between the two collection sites, “site” was a covariate in all linear, linear multivariate and mixed effects models. In all mixed effect models, shoot nested within tree was set as a random effect to account for non-independence of leaves on the same shoot.

To select the best mixed effect model per response, we first chose the optimal variance structure. We built models which included all the explanatory variables but differed in terms of their variance depending on a different explanatory variable. The model with the lowest AIC was then compared against the model with constant variance using likelihood ratio tests. The variance structure that significantly improved model fit was chosen. Next, we assessed the significance of fixed effects. The full model for each response variable was simplified by dropping one explanatory variable or interaction at a time. The change in the model fit was assessed using likelihood ratio tests. Fixed factors that did not improve model fit were dropped from the final model [41]. Significance of the fixed terms in linear models was assessed by computing analyses of variance for the model fit. Model assumptions of all linear and linear mixed effect models were assessed by visually examining plots of residuals against fitted values for the homoscedasticity of residuals, and a Quantile-Quantile plot for the normal distribution of the residuals. Given the limited sample sizes (which could increase the risk of Type II errors), we conducted bootstrap simulations for a subset of response variables to evaluate how increasing the sample size might have affected the results. These simulations were carried out for response variables that showed marginally non-significant (p < 0.2) differences between treatments. All analyses were conducted using R version 3.5.0 [42] and the packages vegan [43], lme4 [44], nlme [45], missMDA [46] and mvabund [47]. Detailed model descriptions are provided in File A in S1 Appendix. Below, we briefly describe the different analyses.

#### Effects of experimental treatments on individual leaf traits

To assess how the experimental treatments affected specific leaf traits, we built mixed effect models for the following response variables: 1) summed concentration of all polyphenolic compounds detected in a leaf, 2) Shannon’s diversity index based on all polyphenolic compounds, 3) concentration of hydrolysable tannins, 4) diversity of hydrolysable tannins, 5) concentration of flavonols, 6) diversity of flavonols, 7) leaf nitrogen content, 8) leaf carbon content, 9) carbon to nitrogen ratio (C:N, log-transformed), 10) LMA and 11) LA (leaf area). Since almost all polyphenol compounds were found in all leaves, the diversity index reflects whether the plant is investing evenly in all of the compounds. Fixed effects in all models were treatment (the five studied leaf types) and collection site (John Krebs field station or Wytham Woods). Additionally, the models on polyphenols included collection date (as Julian date), and all possible two-way interactions as fixed effects. Models on leaf C and N included leaf mass as an additional fixed effect. Models on LMA and LA included percentage of leaf damage and all possible two-way interactions as additional fixed effects.

To investigate the effect of the experimental treatments on the 27 individual polyphenol compounds, we built a linear multivariate model [48–50], in which the matrix of the concentrations of all compounds was modelled as a function of the site, tree, collection date and the treatment.

#### Relationships between leaf traits and leaf gas exchange

To assess the relationship between polyphenol chemistry and photosynthetic rate, we built a mixed effect model with A_1000_ as the response variable and concentration of hydrolysable tannins, diversity of hydrolysable tannins, concentration of flavonols, diversity of flavonols and collection site as explanatory variables. We chose to relate the leaf traits with the photosynthetic parameter A_1000_ because it was estimated for the greatest number of leaves (Fig 1). For each individual polyphenol compound, we investigated the correlation between its concentration and photosynthetic rate (A_1000_), and daytime dark respiration rate (R_d_ [12]) by calculating Pearson’s correlation coefficients. To visualize significant correlations, we built linear models of photosynthetic or respiration rate as a function of the concentration of the specific compound (Fig B and Fig C in S1 Appendix).

To estimate the relationship between photosynthesis and LMA and photosynthesis and LA we built a linear model with A_1000_ as response variable and LMA, LA and site as explanatory variables. To investigate how N and C content was related to photosynthesis, we built a linear model with A_1000_ as response variable and nitrogen content, carbon content and the collection site as explanatory variables.

#### Leaf trait composition

To study how the overall trait composition is influenced by the experimental treatments, we carried out two PCAs. The first PCA included the concentration and diversity of the polyphenol groups (all compounds, hydrolysable tannins, flavonols) and the photosynthetic (A_1000_, A_sat_, V_cmax_, J_max_, TPU, K, R_d_), chemical (C, N, C:N) and physical (LMA, LA) traits. The second PCA included the concentrations of all individual polyphenols but no other traits.

To examine the statistical significance of experimental treatment on leaf trait composition we carried out RDAs with treatment or the tree identity as constraints [51]. To examine the statistical significance of the treatments while accounting for variation between the trees, we carried out a partial RDA where the experimental treatment was set as a constraint, and the tree identity as a condition. We carried out two types of RDAs analogous to the two PCAs. The variation explained by the full model was partitioned to investigate each leaf trait separately.

### Literature survey

To assess whether the polyphenolic compounds detected in our samples have previously been shown to covary with herbivory and/or herbivore performance, we searched in Google Scholar and Web of Science on 25^th^ and 26^th^ June 2018 using the search terms “*compound name*” AND herbivor*. We considered studies that had investigated the concentration of the specific compound 1) in response to experimentally inflicted herbivory, mechanical wounding or application of plant-signalling compounds known to activate anti-herbivore defences, 2) in relation to natural herbivory patterns or 3) in relation to herbivore performance. Only studies that reported results on individual compounds and tested the statistical significance of the studied relationship(s) were included. We estimated the oxidative and protein precipitation activities of the compounds using existing literature [38,39].

## Results

### Does experimentally applied herbivory and mechanical leaf damage change chemical and physical leaf traits?

#### Effects of the experimental treatments and leaf area loss

**Fig 2:**
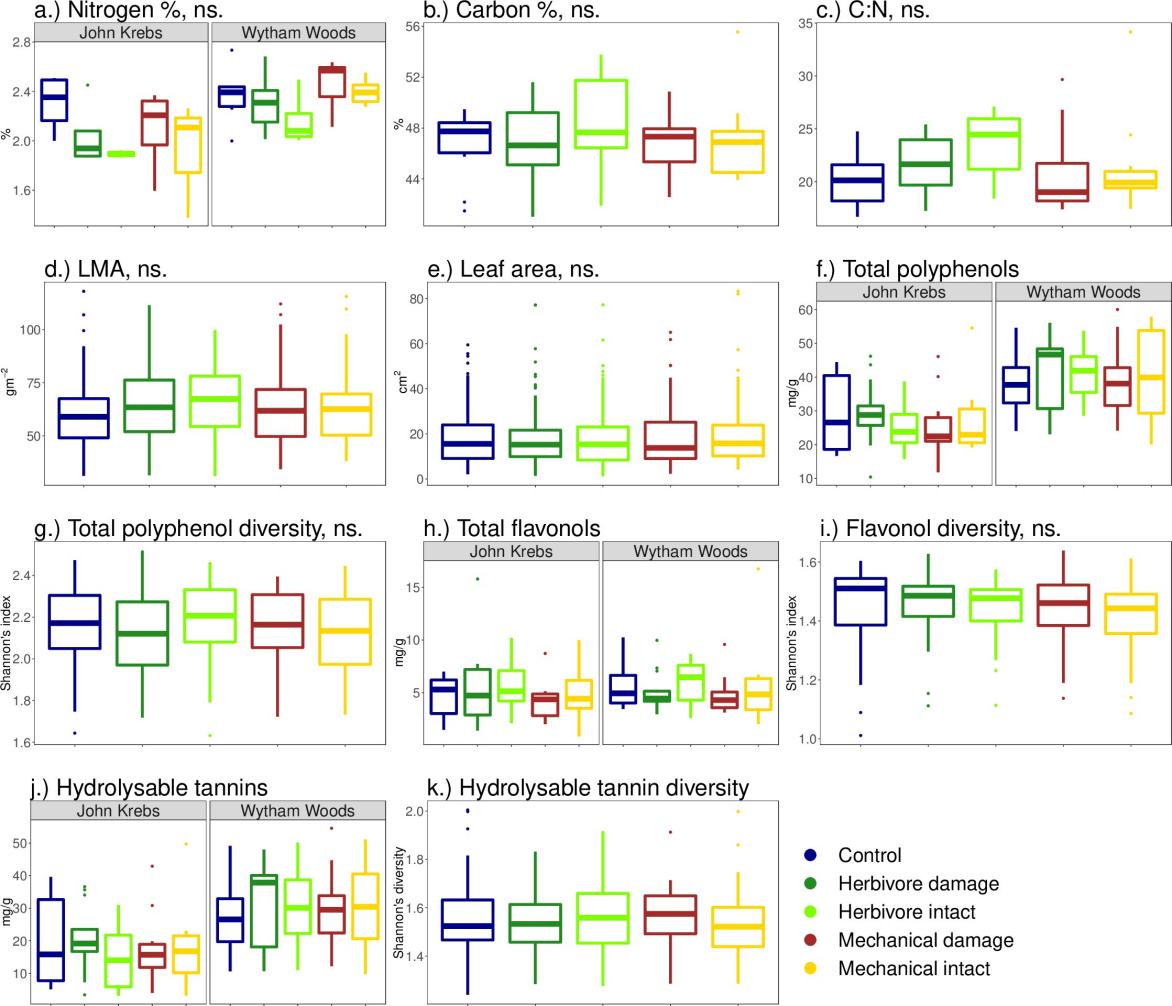
Distribution of leaf traits across experimental treatments. Panel a) shows nitrogen (N) concentration, b) carbon (C) content, c) C:N ratio, d) leaf mass per area (LMA), e) leaf area (LA), f) total concentration of polyphenols, g) Shannon’s diversity for all polyphenols, h) total concentrations of flavonols, i) Shannon’s diversity for flavonols, j) total concentrations of hydrolysable tannins and k) Shannon’s diversity of hydrolysable tannins. Note that for h) and j) the interactions between treatment and collection date, and treatment and site are significant, and for f) and k) the interaction between treatment and collection day is significant. For responses that showed significant difference between the sites, the data is shown for the two sites separately. The line at the middle of the box shows the median, the lower and upper hinges of the box show the 25^th^ to 75^th^ percentile. Data points outside the whiskers are outliers.

For coefficient estimates for models, see Table B in S1 Appendix. There were no clear differences between the experimental treatments in leaf nitrogen (χ^2^ = 7.83, p = 0.10, df = 4, 11, Fig 2A) or carbon content (χ^2^ = 0.98, p = 0.91, df = 4, 9, Fig 2B), C:N ratio (χ^2^ = 5.73, p = 0.22, df = 4, 10, Fig 2C), leaf mass per area (χ^2^ = 7.93, p = 0.09, df = 4, 10, Fig 2D), leaf area (χ^2^ = 6.03, p = 0.20, df = 4, 8, Fig 2E), Shannon’s index for polyphenol diversity (χ^2^ = 1.10, p = 0.89, df = 4, 18, Fig 2G) or Shannon’s index for flavonol diversity (χ^2^ = 7.21, p = 0.13, df = 4, 17, Fig 2I). There were also no differences in concentrations of individual polyphenol compounds between the experimental treatments, although for most compounds, the differences were only marginally non-significant (Table C in S1 Appendix). Simulations to assess whether treatment effects were masked by a small sample size (n = 10 trees) indicated that larger sample sizes (up to 100 trees) would have been unlikely to result in significant differences between the treatments for LM, flavonol diversity and three of the most common polyphenol compounds. For leaf nitrogen, sample sizes of 20 trees or more might have revealed differences between the treatments (Fig F in S1 Appendix).

Experimental treatment had an effect on the total concentration of polyphenols and on the concentrations of hydrolysable tannins and flavonols, but this differed between collection dates and between the two sites (total concentration: treatment × date, χ^2^ = 11.4, p = 0.02, df = 4, 14; hydrolysable tannins: treatment × date, χ^2^ = 39.9, p < 0.001, df = 4, 27; treatment × site, χ^2^ = 32.6, p < 0.001, df = 4, 27; flavonols: treatment × date, χ^2^ = 19.0, p < 0.001, df = 4, 27; treatment × site, χ^2^ = 21.5, p = 0.006, df = 27, 8). The diversity of hydrolysable tannins was affected by treatment, and this effect depended on the collection date (treatment × date, χ^2^ = 12.4, p = 0.01, df = 22, 4).

The proportion of leaf area loss showed a positive relationship with LMA (χ^2^ = 5.06, p = 0.02, df = 1, 6) but no clear relationship with the estimated full leaf area (χ^2^ = 2.32, p = 0.13, df = 1, 5). The leaf area loss was 14.13 + 1.9% per leaf in the herbivory treatment and 10.88 + 1.84% in the mechanical treatment, close to natural levels of herbivory in the area (8.45 + 0.39; see [12]).

#### Differences in leaf traits between sites and collection days

Trees in Wytham Woods had higher nitrogen content (χ^2^ = 4.18, p = 0.04, df = 1,7) and higher total polyphenol concentration (χ^2^ = 3.87, p = 0.049, df = 1, 6) than trees at the John Krebs field station. The concentrations of many individual polyphenol compounds differed between the two sites (Table C in S1 Appendix), with majority of compounds (19 out of 27) having higher concentration in Wytham Woods.

The diversity of all polyphenols increased over the measuring period (χ^2^ = 11.02, p < 0.001, df = 1, 14, Table B in S1 Appendix). The concentrations of all individual polyphenol compounds were also affected by the leaf collection date (Table C in S1 Appendix), with most compounds (16 out of 27) decreasing in concentration over the season.

### Are changes in leaf gas exchange linked to parallel changes in other leaf traits?

#### Relationships between the different leaf traits

**Fig 3:**
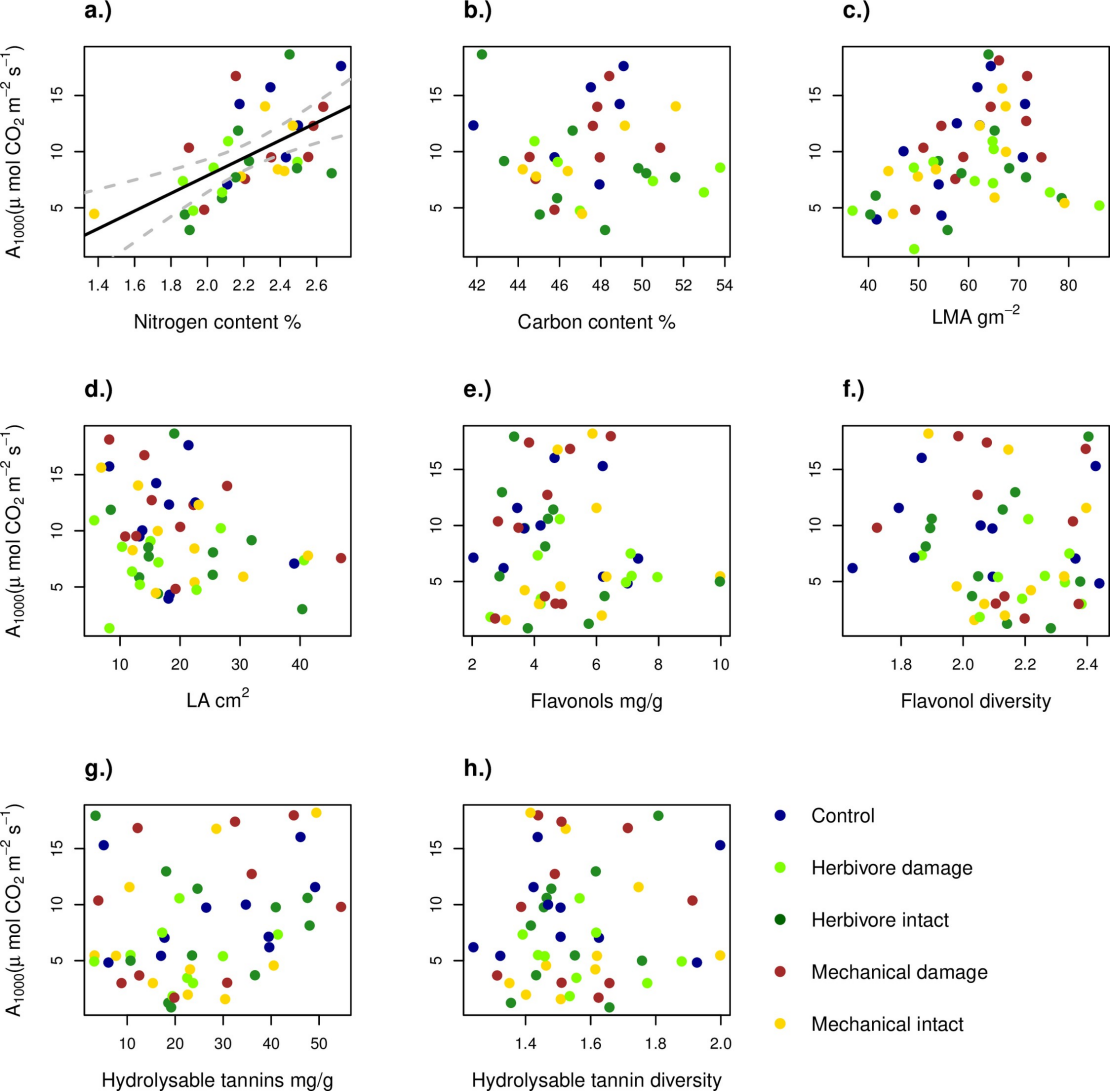
The relationship between photosynthetic rate (A_1000_) and the studied leaf traits. Panel a) shows photosynthesis rate and nitrogen (N) concentration b) carbon (C) content, c) leaf mass per area (LMA) d) leaf area (LA), e) total concentration of all flavonols, f) Shannon’s diversity for flavonols, g) total concentrations of hydrolysable tannins and h) Shannon’s diversity of hydrolysable tannins in the different experimental treatments. Note that for panels a-d the relationship represents values *per tree*, because photosynthetic rate and nutrient content were measured on different leaves. Only the relationship in panel a) is statistically significant.

Photosynthetic rate was positively related to leaf nitrogen content (F = 8.16, p = 0.007, df = 1, 32, Fig 3A). There was no significant relationship between photosynthesis and carbon content (F = 0.03, p = 0.87, df = 1, 31, Fig 3B), LMA (F = 3.53, p = 0.07, df = 1, 42, Fig 3C), leaf area (F = 0.10, p = 0.75, df = 1, 42, Fig 3D), flavonol concentration (χ^2^ = 3.38, p = 0.07, df = 1, 6, Fig 3E), flavonol diversity (χ^2^ = 0.13, p = 0.71, df = 1, 6, Fig 3F), hydrolysable tannin concentration (χ^2^ = 0.06, p = 0.81, df = 1, 6, Fig 3G) or hydrolysable tannin diversity (χ^2^ = 0.48, p = 0.49, df = 1, 6, Fig 3H).

Of the individual polyphenols, four hydrolysable tannins (cocciferin D_2_, vescavaloninic acid, tellimagrandin I, galloyl-HHDP-glucose) and one flavonol (quercetin diglycocide) correlated positively with photosynthesis (Fig A in S1 Appendix). Chlorogenic acid correlated negatively with photosynthesis (Fig A in S1 Appendix). Three hydrolysable tannins (monogalloylglucose, vescalagin, castalagin) correlated negatively with leaf respiration rate (measured at the tree level), wheres two flavonols (quercetin glucuronide, kaempferol diglycoside) correlated positively with respiration rate (Fig B in S1 Appendix).

#### Differences in leaf trait composition between treatments and trees

**Fig 4:**
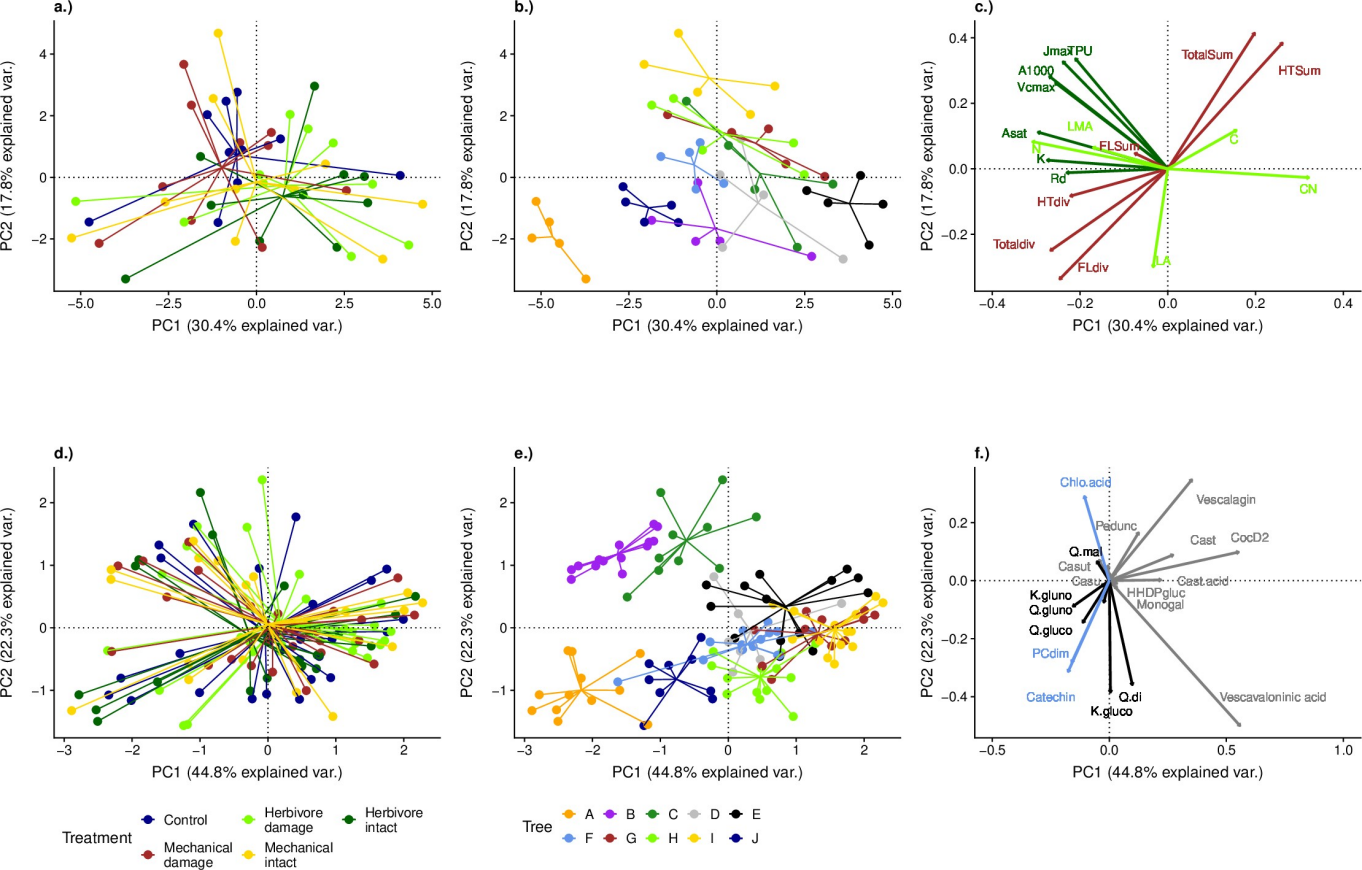
**Results from principal component analyses (PCA) on studied leaf traits (excluding the concentrations of individual polyphenol compounds; panels a-c, see Table 1), and on polyphenol composition based on individual compounds (panels d-f, see Table A in** S1 **Appendix**). Results are grouped either by experimental treatment (panels a and d) or by study tree (panels b and e). Data points in panels a-b represent averages per tree and treatment. In panels d-e each data point is one leaf. Panels c) and f) show the relationships between different leaf traits. In panel c) dark green arrows represent photosynthetic parameters, brown arrows represent traits reflecting polyphenol chemistry, and light green arrows other traits. In panel f) black arrows show hydrolysable tannins, grey arrows show flavonols and blue arrows show other chemical compounds (see Table A in S1 Appendix). Note that respiration rate has been made positive to make the graph more intuitive (i.e. so that larger respiration values correspond to higher respiration rate). See Fig C in S1 Appendix for traits determining the PC axes.

**Fig 5:**
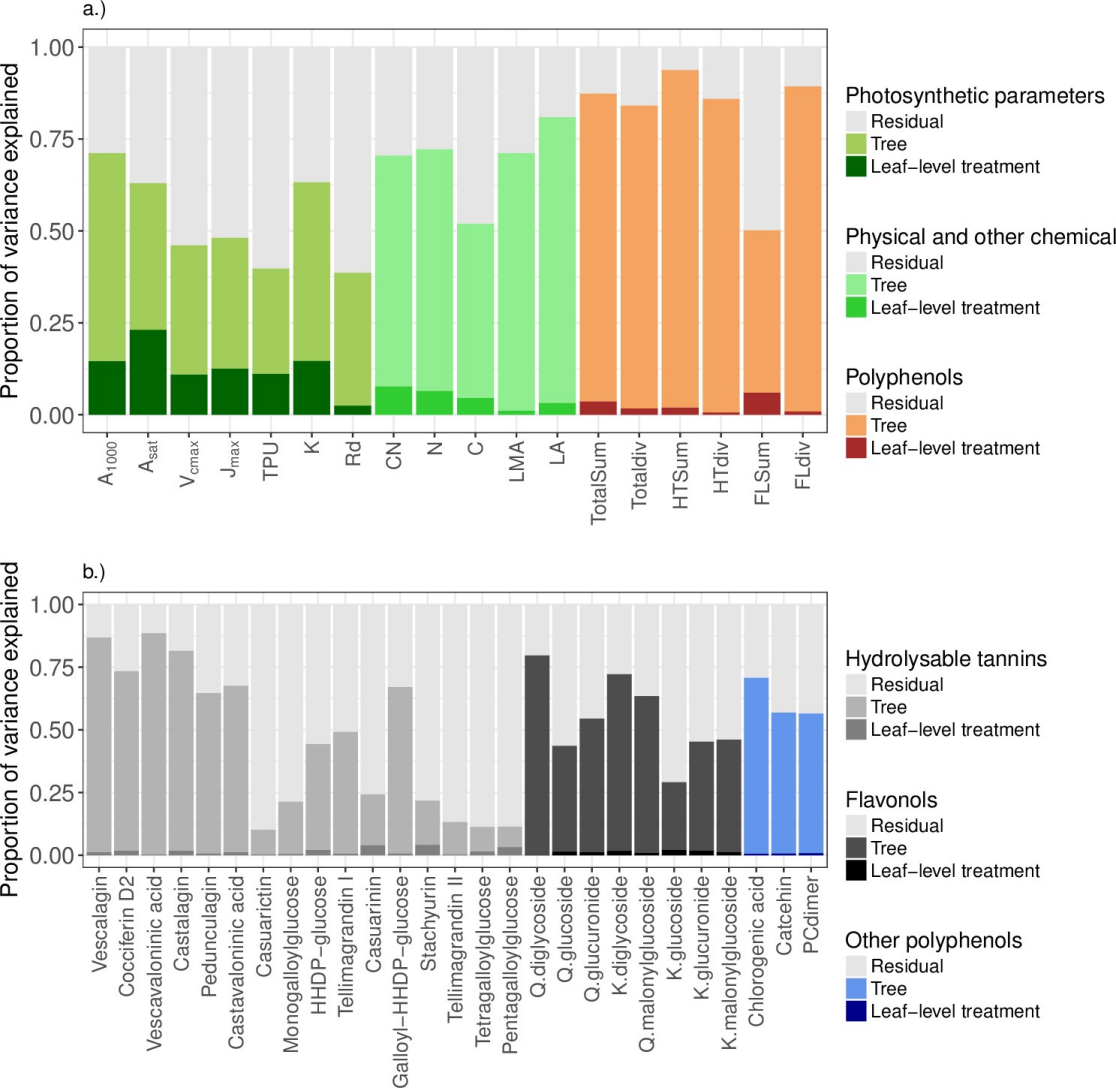
**The proportion of variance in each leaf trait explained by the experimental treatment (dark colours), tree (light colours), and the unexplained variation (light grey bars) based on partitioned variance from the partial redundancy analysis (RDA).** Panel a.) shows photosynthetic traits (dark green), physical and non-defensive chemical traits (lighter green) and concentrations and diversities of all polyphenols and the two largest polyphenol groups (hydrolysable tannins and flavonols, orange). Panel b.) shows variation in individual polyphenols, grouped as hydrolysable tannins (light greys), flavonols (dark greys) and other compounds (blues). Within each group, the compounds are shown in order of decreasing abundance.

The results from the PCAs showed no differences in the composition of the studied leaf traits (concentration and diversity of polyphenol groups, photosynthetic parameters, C and N content and physical leaf traits; F = 1.18, p = 0.26, df = 4, 45, Fig 4A) or in the composition of polyphenol compounds (F = 0.36, p = 0.99, df = 4, 114, Fig 4D) between the treatments. Tree identity had a significant effect on overall composition of leaf traits (F = 5.01, p = 0.001, df = 9, 40, Fig 4B) and on polyphenol composition (F = 24.62, p = 0.001, df = 9, 109, Fig 4E). The largest portion of variation in all traits was explained by the variation between individual trees (Fig 5).

After accounting for the variation between the study trees, the partial RDA revealed differences between the treatments in the compostion of leaf traits (with polyphenol chemistry expressed as summed metrics rather than as concentrations of individual compounds; Fig D in S1 Appendix, F = 2.28, p = 0.004, df = 4, 36). The intact and damaged leaves in the herbivory treatment differed from the rest of the leaves along the first RD axis (Fig D in S1 Appendix), which was mainly influenced by the photosynthetic parameters (Fig D in S1 Appendix). The polyphenol composition (assessed as the concentrations of individual compounds) did not differ between the treatments even after accounting for differences between the trees (F = 0.97, p = 0.47 df = 4, 105).

The concentration of polyphenols correlated negatively with the diversity of polyphenols (Pearson's r = −0.67, t = −9.81, df = 117, p < 0.001, Fig 4C, Fig E in S1 Appendix), and with the size of the leaf (Pearson's r = −0.47, t = −3.66, df = 46, p < 0.001, Fig 4C, Fig E in S1 Appendix).

### Does the published literature provide evidence for the measured polyphenols functioning as anti-herbivore defences?

The literature revealed a varying nature of the relationship between herbivory and plant polyphenol concentration: most compounds have been observed to function both as defences and attractants or to have no relationship to herbivory at all. We found slightly less evidence for anti-herbivore activity for compounds that ranked low on oxidative or protein precipitation activity, though these trends were not significant. For details, see File C in S1 Appendix.

## Discussion

In this study, we tested whether experimentally manipulated herbivory and mechanical leaf damage affect a number of chemical and structural leaf traits, whether these traits correlate with photosynthesis, and whether leaf chemistry correlates with leaf respiration. We measured concentrations of 27 polyphenol compounds, leaf nitrogen and carbon content, leaf mass per area and leaf area. We also surveyed the literature on previously recorded relationships between herbivory and the polyphenol compounds observed in this study. We detected no differences in the concentrations of individual polyphenol compounds, nitrogen or carbon content, leaf mass per area or leaf area between the experimental treatments. While we detected some effects of the experimental treatments on the overall polyphenol concentration, the concentration and diversity of hydrolysable tannins and the concentration of flavonols, these varied between the two study sites and/or depended on the timing of leaf collection. Leaf nitrogen content and five individual polyphenol compounds correlated positively with photosynthesis. Respiration was related negatively with three compounds and positively with two. There was large variation among tree individuals in their trait composition, especially in terms of their polyphenol composition. Below, we discuss these results in detail.

### No clear changes in leaf traits following herbivory and mechanical leaf damage, but large variation in trait composition between trees and sites

We predicted that herbivory and/or mechanical damage would result in changes in the concentration, diversity or composition of polyphenols, a larger leaf mass per area and/or result in leaves growing smaller. However, there were no clear differences in the studied leaf traits between experimental treatments. Experimental treatments had a significant effect on total polyphenol, hydrolysable tannin and flavonol concentrations, but this effect depended on the study site and collection date (i.e. a significant site × treatment and date × treatment interactions).

To investigate further how the effect of treatment and date on polyphenol concentration differed between the two sites, we conducted *post hoc* analyses assessing the effect of treatment on polyphenol chemistry for the two sites separately. Site-specific linear mixed effects models revealed that the effect of treatment on the concentration of flavonols and hydrolysable tannins differed between the collection days at only one of the sites (Wytham Woods). At the other site (John Krebs field station), treatment alone had a significant effect on hydrolysable tannin concentration: the leaves damaged by herbivores had a higher hydrolysable tannin concentration than intact leaves growing on the same shoot (on average 20.4 mg/g ± 2.9 SEM compared to 14.7 mg/g ± 3.0 SEM). See File B in S1 Appendix for further details on the site-specific models.

Previous studies on oak have found increased concentrations of hydrolysable tannins after insect defoliation [16] or experimental induction with jasmonic acid [29]. These types of changes in plant chemistry are often thought to result from herbivory-induced defence reactions in the host tree [3]. Nevertheless, several studies show no changes in plant chemistry after herbivory [52,53]. In line with this, the published literature reveals few consistent relationships between herbivory and the 27 polyphenol compounds measured in this study: our literature survey suggests that most compounds seem to function both as defences and attractants or to have no relationship to herbivory at all (see File C in S1 Appendix). The effects of defensive compounds on herbivores often depend on the overall chemical or nutritional content of the plant, so that the presence of one compound can enhance or suppress the effect of the other [30,54–56]. Synergic or antagonistic effects are thought to be common [55,57], but so far only a few studies have tested for them [54–56,58]. The inconsistent patterns detected in the literature survey might be due to the specificity of plant-herbivory interactions: the outcome can depend on the species or genotype of the two interacting parties [28,59], on which other chemicals are present and in what quantities [54–56], or on the chemistry of the insect itself [60].

Many studies have shown high variation in plant chemistry between individuals of the same species [29,36,61–64] or between parts of the same individual [25,65]. The high level of variation in leaf chemistry might arise because the concentration of many secondary chemicals depends on several factors other than herbivory such as temperature [54,66], soil nutrients [67], light [68,69], presence of pathogens [70,71] or competitors [72,73]. There are several factors that differed between the two experimental sites, which might explain the observed variation in polyphenol chemistry. The study trees at Wytham Woods were old (150–200 years) and the leaves collected from them were upper canopy sun leaves with higher nitrogen content. The trees measured by the John Krebs field station were young (33/34 years at the time of the study), planted, and the leaves collected from them came from the lower branches, though still exposed to the sun. Within the site by the field station, three of the trees (A-C, see Fig 3B and 3E) experienced intense sunlight for several hours during the day, whereas two of the trees (D and E, see Fig 3B and 3E) were shaded during the warmest times of the day. These differences in growth locations most likely contributed to the differences in leaf traits and chemical composition between trees and/or between shoots within a tree.

We found that the effects of the treatments on the summed polyphenol concentration, on the concentration and diversity of hydrolysable tannins and on the concentration of flavonols also depended on the timing of the collection of leaf samples (see File B in S1 Appendix for details). The seasonal variation in oak leaf chemistry [26,27] might have altered how the treatments were affecting the measured chemical traits. If for example the concentration of hydrolysable tannins was changing over the period of leaf sample collection, the differences in their concentration between the treatments might be less visible on leaves collected during certain times of the season. Future studies should aim to account for seasonal changes in chemistry for example by restricting leaf collection to a specific time, or by collecting leaves systematically throughout the season.

Since we found no differences in polyphenol chemistry between the treatments, the reduced photosynthetic rate is unlikely to be due to changes in resource allocation between growth and defence. Instead, herbivory might have affected the onset of leaf senescence [74,75], which could have decreased photosynthesis. Photosynthesis could also have been reduced due to “auto-toxicity”, if constitutively stored defence compounds were activated after herbivory, damaging the photosynthetic machinery [75,76].

### Correlation between leaf gas exchange and leaf traits

We found that nitrogen was positively related to photosynthesis, that five different polyphenols (cocciferin D_2_, vescavaloninic acid, quercetin diglycoside, tellimagrandin I, galloyl-HHDP-glucose) correlated positively with photosynthesis, and that chlorogenic acid correlated negatively with photosynthesis. A positive relationship between nitrogen and photosynthesis is commonly observed because many of the enzymes needed for carbon-fixing contain a large portion of the leaf nitrogen [4]. Two compounds (quercetin glucuronide, kaempferol diglycoside) correlated positively with leaf respiration rate, and three compounds (vescalagin, castalagin, monogalloylglucose) negatively.

Based on the negative effects defence reactions can have on photosynthesis [5,6], we expected to find a negative correlation between photosynthetic rate and polyphenol chemistry. Nevertheless, in our study, most individual compounds and their summed metrics did not correlate with photosynthesis, and of the significant correlations, all but one were positive. Positive correlations between photosynthesis and leaf chemicals might not be surprising, if photosynthetic products are needed for the formation of secondary metabolites [77,78] and if these metabolites are not related to leaf-level induced defence reactions. The concentration of many secondary metabolites, including hydrolysable tannins, depend at least partly on the available carbohydrate pool [52,77]. The polyphenols studied here might function as constitutive rather than induced defences, and for example be seasonally produced when herbivory pressure is highest [26,27], regardless of changes in leaf-level damage. Alternatively, the chemical compounds induced early in the season might not have been present at the time of leaf collection [79]. Several studies have also shown that the growth-defence trade-off might not be caused by simple resource allocation, but arise through prioritization of one process over the other, depending on the environment [10,80,81]. Thus, the trade-off might be detectable only under certain conditions, for example if the lack of certain nutrients is limiting both processes, or if plants are actively competing against each other [73,80,81]. Relative allocations to growth and defence might also differ between perennial and annual plants, if, for example, regrowth or increased investment in photosynthesis in the future benefits the plant more than inducing an immediate defence reaction [82,83].

### Conclusions

We tested whether leaf-level manipulation of herbivory or mechanical damage changes chemical and structural leaf traits, and whether these traits correlate with photosynthesis and leaf respiration rates. The experimental manipulations did not induce any changes in leaf structure or nitrogen and carbon content. Leaf polyphenol chemistry was affected by the treatments, but differently depending on the study site and time of the year. Most polyphenols did not correlate with photosynthesis or correlated positively, opposite of what would be expected if photosynthesis was suppressed by changes in defensive chemistry. We suggest that the results of the field experiment are due to small-scale variation in environmental conditions experienced by the host tree. Leaf chemistry is often affected by microclimate, nutrient availability and intensity of competition from surrounding plants. All these factors differed between the two study sites and might have had a stronger effect on plant chemistry than leaf-level changes in herbivory or mechanical damage. Based on this study, herbivore-induced leaf-level changes in photosynthesis cannot be explained by concurrent changes in polyphenol chemistry or leaf traits.

## Supporting information

S1 Appendix Supporting information file including all supporting material. (DOCX)
