# Supplementary material for: Changes in oak (Quercus robur) photosynthesis after winter moth (Operophtera brumata) herbivory are not explained by changes in chemical or structural leaf traits
Source: PLoS One. 2020 Jan 24;15(1):e0228157. doi: 10.1371/journal.pone.0228157 (PMC6980561; doi:10.1371/journal.pone.0228157)
Supplement: S1 Appendix — (DOCX) [file pone.0228157.s001.docx]

**Supporting information for Visakorpi K., Riutta T., Malhi Y., Salminen J-P., Salinas N and Gripenberg S. 2020. Changes in oak (*Quercus robur*) photosynthesis after winter moth (*Operophtera brumata*) herbivory are not explained by changes in chemical or structural leaf traits.**

**File A. Detailed methods**

**Collecting leaf traits.** We measured concentrations of individual polyphenol compounds, leaf nitrogen (N) and leaf carbon (C) content, leaf mass per area (LMA) and leaf area without herbivory (LA), and used previously measured leaf gas exchange traits [1]. The sampling times of the chemical and structural traits were determined by when the gas exchange measurements were taken and were thus unevenly spread throughout the season. All samples were collected after the peak canopy leaf area, and maximum photosynthetic capacity (see [2]) was reached, but before senescence. However, measuring temporal variation in leaf traits was not our main goal.

Leaves from shoots that were used for photosynthetic measurements (three out of the 15 shoots per tree) were collected within few days after the final photosynthesis measurement on that tree had taken place (11^th^ July to 11^th^ August 2016). This was done to ensure that the timing of the leaf collection would match the timing of the measured photosynthesis without disturbing the further photosynthetic measurements of that tree. The remaining leaves were collected at the end of the measuring period on 12^th^ August 2016. Leaves were transported in a coolbag to a freezer and freeze-dried within few weeks of collection. In total, we measured polyphenol content for 119 leaves from 74 shoots. Of the remaining experimental leaves not used for polyphenol and photosynthetic measurements, 54 leaves from 36 shoots from 10 trees were analysed for carbon and nitrogen content. Leaves from the remaining 39 shoots were stored for potential future analyses. All collected leaves were sun leaves, either from upper canopy (at the site in Wytham Woods) or from lower branches exposed to the sun (at the site by the John Krebs field station).

Analyses of the leaf carbon and nitrogen content were carried out in the Pontificia Universidad Católica del Perú. Dry leaf material was ground to fine powder in a variable speed mini cutting mill, 115/230V (Eberbach), and C and N content (%) was determined by combustion analysis using LECO’s TruMac C:N analyser.

Analyses of the polyphenol concentrations were carried out in the laboratory of the Natural Chemistry Research Group at the University of Turku, Finland, using ultra-performance liquid chromatography high-resolution mass spectrometry (UPLC-HR-MS). Briefly, 20 mg of the dry plant powder was extracted 2 x 3 h with 1.4 mL acetone/water (80:20, v:v). Acetone was evaporated from the combined extract by the Eppendorf concentrator and the water phase was freeze-dried. The lyophilized water phase was dissolved in 1 mL ultra-pure water and filtered via 0.2 µm PTFE filter. The UPLC-Orbitrap-MS analyses of individual polyphenols were acquired from the 5-time dilution of this filtered water phase. The UPLC system consisted of a binary solvent manager, sample manager, column (Acquity UPLC BEH Phenyl, 30 mm × 2.1 mm i.d., 1.7 µm, Waters Corporation), and photodiode array detector (Acquity UPLC®, Waters Corporation, Milford, MA, USA) coupled to a hybrid quadrupole-Orbitrap mass spectrometer (Q Exactive™, Thermo Fisher Scientific GmbH, Bremen, Germany; [3]). The compounds were quantified from the full scan trace by monitoring the peak areas of the most prominent ions (typically [M-H]^-^ or [M-2H]^2-^) of each compound and using external calibration curves to convert peak areas into mg/ml concentrations. For each sample, we obtained detailed estimates of the concentration of 27 polyphenol compounds (Table A in S1 Appendix).

To calculate leaf mass per area (LMA, g/m^2^) and leaf area (LA, cm^2^), we used leaf material collected at the end of the 2015 field season (103 shoots, 616 leaves; collected 30^th^ October – 2^nd^ November 2015). Leaves were scanned and dried in the oven (70°C, 72h) and the remaining and original leaf area were estimated using ImageJ software (NIH, MD, USA). Dried samples were weighted to determine LMA. A small number of control shoots that had contained damaged leaves (n = 14) were discarded from further analyses.

**Detailed model descriptions**

*Treatment effects on polyphenol chemistry.*

To study the effect of the experimental treatments on polyphenol chemistry, we built mixed effect models with either the summed concentration of all polyphenolic compounds detected in a leaf, Shannon’s diversity index based on all compounds, or the concentration or diversity of each of our two focal polyphenol subgroups (hydrolysable tannins and flavonols) as a response. Fixed effects were treatment (the five studied leaf types), the collection site (John Krebs field station or Wytham Woods), collection date (as Julian date), and all possible two-way interactions. For models for hydrolysable tannin and flavonol concentration, diversity of all compounds, diversity of flavonols and diversity of hydrolysable tannins, variance structure was allowed to vary between the tree individuals to account for unequal variances between trees. Random effects were shoot ID nested within tree ID.

To investigate the effect of the experimental treatments on the 27 individual polyphenol compounds, we built a linear multivariate model [4–6], in which the matrix of all compounds was modelled as a function of the site, tree, collection date and the treatment. We then extracted the univariate test statistics for each compound separately.

*Treatment effects on leaf carbon and nitrogen*

To investigate the effect of treatment on leaf carbon and nitrogen content, we built a linear mixed effect model of either per-leaf nitrogen or carbon content or the log-transformed relationship between the two (C:N) as a function of treatment, the collection site and leaf mass. Interactions were omitted to avoid spurious interactions due to small sample size (n = 36, [7]). For the models for nitrogen content and C:N ratio, variance was set to vary to the power of the covariate leaf mass to improve the distribution of the model residuals. Random effects were shoot ID nested within tree ID.

*Treatment effects on leaf mass per area and leaf area*

To investigate the effect of the experimental treatment on LMA, we built a linear mixed effect model of LMA (as gm^−2^) as a function of treatment, collection site, percentage of leaf damage and all two-way interactions. After carrying out model selection and examining model residuals against fitted values, we included leaf dry mass in the model as an explanatory variable to reduce heteroscedasticity of the model residuals. Shoot nested within tree was set as a random effect.

To investigate whether herbivory affected leaf size (as could be expected if trade-offs between growth and defence were present), we tested the effect of the treatment on LA (i.e. the estimated leaf area in the absence of any damage). We built a linear mixed effect model of the estimated leaf area as a function of the experimental treatment, collection site, the percentage of leaf damage, and all possible two-way interactions. Variance structure was allowed to vary between the tree individuals to account for unequal variances between trees. Random effect was shoot ID nested within tree ID.

*Relationships between summed metrics of polyphenol chemistry and photosynthesis*

To study the relationship between polyphenol chemistry and photosynthetic rate, we built a linear mixed effect model with photosynthetic rate (A_1000_) as the response variable and concentration of hydrolysable tannins, diversity of hydrolysable tannins, concentration of flavonols, diversity of flavonols and the collection site as explanatory variables. Since A_1000_ was measured on the same leaves that were later collected for chemical analyses, we were able to build the models using leaf-level data. Total concentration of polyphenols and diversity of all polyphenols were not included in the model due to collinearity with the explanatory variables (i.e. concentrations and diversities of the two polyphenol subgroups). No interactions were included, because the possible two-way interaction combinations (interactions between the two polyphenol groups, or between polyphenol concentration and diversity) were not biologically relevant. Random effect was shoot ID nested within tree ID.

*Relationships between individual polyphenols and photosynthesis*

For each individual polyphenol compound, we investigated the correlation between its concentration and both A_1000_ and daytime dark respiration rate (R_d,_ estimated from the photosynthesis-light response curves, [1]). Since respiration was measured from different leaves and during a different season than polyphenol content, we calculated average values for each treatment in each tree and used these tree/treatment specific averages (n = 10 per leaf-level treatment) in the analyses. We calculated Pearson’s correlation coefficient for each pairwise compound-rate comparison. To visualize significant correlations, we built linear models of photosynthetic or respiration rate as a function of the concentration of the specific compound and plotted the model predictions (Fig B and Fig C in S1 Appendix).

*Relationship between LMA and photosynthesis and LA and photosynthesis*

To estimate the relationship with photosynthesis and LMA and photosynthesis and LA we built a linear model with A_1000_ as response variable and LMA, LA (as tree/treatment specific averages) and site as explanatory variables. No interactions were included to avoid spurious interactions due to small (n = 50) sample size [7].

*Relationship between leaf C/N content and photosynthesis*

To investigate how N and C content was related to photosynthesis, we built a linear model with A_1000_ as response variable and nitrogen content, carbon content and the collection site as explanatory variables as tree/treatment -specific averages. Due to collinearity, C:N ratio was not included in the model. No interactions were included to avoid spurious interactions due to small (n = 50) sample size [7].

*Leaf trait composition*

To study how the individual leaf traits correlate with each other and whether they are influenced by the experimental treatments, we performed a PCA. To investigate changes in all measured leaf traits separately from changes in the polyphenol composition only, we carried out two PCAs. The first PCA included the concentration and diversity of the polyphenol groups (all compounds, hydrolysable tannins, flavonols) and the photosynthetic (A_1000_, A_sat_, V_cmax_, J_max_, TPU, K, R_d_, see main text and [1]), chemical (C, N, C:N) and physical (LMA, LA) traits. The second PCA included the concentrations of all individual polyphenols but no other traits. The first PCA was constructed using tree/treatment specific averages, whereas the second PCA was constructed using leaf-level data.

The first PCA (all traits) was carried out as an iterative PCA [8]. This was done in order to include carbon and nitrogen content, the sample size of which was smaller than that of the other traits. This method has been shown to perform well on different datasets with missing values [8]. We considered values missing at random [9]. Data were log-transformed, scaled and centred to improve the ordination results [10]. For the second PCA (individual polyphenols only), the data were log-transformed and centred to improve the visualization of the ordination results. The data were plotted on the first five PC axis, and the resulting ordination plots were examined visually to identify clustering around treatment groups, or around the experimental trees.

To examine the statistical significance of the experimental treatment on the leaf trait composition we carried out RDAs, in which the treatment or the tree identity were set as “constraints” [11]. To examine the statistical significance of the treatments while accounting for variation between the trees, we carried out a partial redundancy analysis (pRDA) where the experimental treatment was set as a constraint, and the tree identity as a condition. Partial RDA removes the variation caused by the conditioned variable, i.e. in this case the variation between the individual trees. We carried out two types of RDAs similar to the two PCAs: one for the concentration and diversity of the summed polyphenol groups (all compounds, hydrolysable tannins, flavonols), the photosynthetic (A_1000_, A_sat_, V_cmax_, J_max_, TPU, K, R_d_), chemical (C, N, C:N) and physical (LMA, LA) traits, and another for individual polyphenol compounds only. The statistical significance of the constrained variables was determined by examining the amount of constrained vs. unconstrained variation revealed by the models (constrained variation referring to variation explained by the explanatory variable treatment or tree ID). The significance was tested with permutation tests (n = 999 permutations). The variation explained by the full model was then partitioned to investigate each leaf trait separately.

*Simulation for assessing the effect of the small sample size*

To estimate whether the non-significant effect of treatment on some of the response variables was due to the small sample size (n = 10 trees), we re-sampled the data through bootstrapping, with different sample sizes (n = ranging from 10 to 100). We then ran a linear model with treatment as the only explanatory variable through each bootstrapped dataset. For each of the different sample sizes, we ran the model 1000 times. We then calculated, for each sample size, the proportion of models with a significant (p < 0.05) treatment effect. The simulations were carried for the datasets in which leaf treatment was marginally non-significant (N, p= 0.1; LMA, p = 0.09; flavonol diversity, p = 0.13), and therefore most likely candidates for Type II error. Since most of the individual polyphenol compounds showed marginally non-significant differences between the treatments (see Table C in S1 Appendix), we ran the simulation only for the three most abundant compounds (cocciferin D_2_, p = 0.06; vescalagin, p = 0.08; catechin, p = 0.09).

**File B. Additional analyses**

*The interaction between treatment and site*

To study the interaction between the experimental treatments and site affecting leaf chemistry, we built linear mixed effect models for both sites (Wytham Woods and John Krebs field station) separately with the summed concentration of flavonols or hydrolysable tannins as a function of the collection date, treatment, and their interactions (Table B.1 in File B in S1 Appendix). Variance was set to vary between the trees for both models on flavonol concentration, and on the model on hydrolysable tannin concentration for Wytham Woods.

Flavonol concentration. In Wytham Woods, the flavonol concentration was significantly affected by the interaction between date and treatment (χ^2^ = 22.8, p < 0.001, df = 17, 4, Fig B1a in File B in S1 Appendix). By John Krebs field station, there were no significant effect of the date (χ^2^ = 0.03, p = 0.87, df = 13, 1), the treatment (χ^2^ = 1.31, p = 0.86, df = 13, 4) or their interaction (χ^2^ = 2.10, p = 0.72, df = 17, 4, Fig B1b in File B in S1 Appendix) on flavonol concentration.

Hydrolysable tannin concentration. In Wytham Woods, the interaction between date and treatment had a significant effect on hydrolysable tannin concentration (χ^2^ = 39.8, p < 0.001, df = 4, 17, Fig B1c in File B in S1 Appendix). On leaves collected by the John Krebs field station, the hydrolysable tannin concentration was significantly different between the treatments (χ^2^ = 13.6, p = 0.008, df = 4, 8, Fig B1d in File B in S1 Appendix). The significant differences were between intact and damaged leaf in the herbivory treatment (t.ratio = 3.21, p = 0.03). Collection date had no effect on hydrolysable tannin concentration at the field site (χ^2^ = 0.02, p = 0.88, df = 1, 9), nor did the interaction between date and treatment (χ^2^ = 9.18, p = 0.06, df = 4, 13, Fig B1d in File B in S1 Appendix).

*The interaction between treatment and collection date*

Since assessing seasonal changes in leaf chemistry was not one of our study questions (and since we did not want to cause additional disturbance to the trees until all physiological leaf measurements had been taken), leaf samples were not collected at multiple times over the growing season. Instead, half of the leaves were collected simultaneously with the photosynthetic measurements, and rest at the end of the growing season. The unequal spread of the samples over the season limits our ability to investigate the interactive effects between herbivory and the collection date. Nevertheless, to study the significant interaction between treatment and collection date affecting leaf chemistry in more detail, we built linear mixed effect models for early and late-season leaves separately with concentration of hydrolysable tannins, diversity of hydrolysable tannins, or concentration of flavonols (i.e. response variables that showed significant date × treatment interaction, Table B1 in File B in S1 Appendix) as a function of the study site, collection date (for early season model only) and the experimental treatment. Interactions were not included in the early-season model to avoid spurious interactions due to small sample size. Interaction between site and treatment was included in the model for late-season leaves. For the late-season model on hydrolysable tannin concentration, the variance was allowed to be different between the two sites. For early-season model on hydrolysable tannin diversity, the variance varied exponentially with the collection date. For late-season model on flavonol concentration, the variance varied between the experimental treatments. When treatment turned out significant, we performed Tukey’s test to find out which treatments differed from each other. Model selection and diagnostics checks were performed as described in the main text (see “Statistical analyses”).

Hydrolysable tannin concentration. For late-season leaves, the hydrolysable tannin concentration was affected by the experimental treatment (χ^2^ = 17.3, p = 0.002, df = 4, 9). The significant differences were between control leaves and leaves damages by herbivores (t.ratio = −3.16, p = 0.03) and between intact and damaged leaves in the herbivory treatment (t.ratio = 3.93, p = 0.006). There were no differences between the two sites (χ^2^ = 1.80, p = 0.18, df = 1, 10) in hydrolysable tannin concentration on late-season leaves. For leaves collected throughout the season, only collection date had a significant effect (χ^2^ = 5.65, p = 0.02, df = 1, 5), with no differences between sites (χ^2^ = 0.03, p = 0.85, df = 1, 6) or treatments (χ^2^ = 4.52, p = 0.34, df = 4, 9).

Hydrolysable tannin diversity. There was no effect of treatment (χ^2^ = 7.31, p = 0.12, df = 4, 9), site (χ^2^ = 0.59, p = 0.44, df = 1, 6) or date (χ^2^ = 1.08, p = 0.30. df = 1, 6) on the diversity of hydrolysable tannins on leaves collected throughout the season. Similarly, there was no effect of treatment (χ^2^ = 7.56, p = 0.11, df = 4, 8), site (χ^2^ = 0.03, p = 0.87, df = 1, 5) or their interaction (χ^2^ = 6.58, p = 0.16, df = 4, 13) on the hydrolysable tannin diversity on leaves collected at the end of the growing season.

Flavonol concentration. There was no effect of treatment (χ^2^= 1.32, p = 0.86, df = 4, 8), site (χ^2^ = 0.88, p = 0.35 df = 1, 6) or date (χ^2^ = 0.42, p = 0.52. df = 1, 5) on the concentration of flavonols on leaves collected throughout the season. Similarly, treatment (χ^2^ = 3.77, p = 0.44, df = 4, 12) or site (χ^2^ = 0.32, p = 0.57, df = 1, 9) or their interaction (χ^2^ = 2.94, p = 0.57, df = 4, 17) had no effect on late-season flavonol concentration.

**Table B1.** The significant explanatory factors for the linear mixed effect models testing the effect of the leaf level treatments on leaf chemistry at two sites and at two temporal scales separately. The response variables had previously been identified to have been significantly affected by the interaction between collection date and treatment and between site and treatment (concentration of hydrolysable tannins and flavonols). ~1 refers to a null model, i.e. none of the explanatory factors were significant. HT = hydrolysable tannins, FL= flavonols.

| **Response** | **Wytham Woods** | **Field station** | **During the season** | **At the end of the season** | |
| --- | --- | --- | --- | --- | --- |
| Concentration of HT | Date * Treatment | Treatment | Date | Treatment |  |
| Concentration of FL | Date * Treatment | ~ 1 | ~ 1 | ~ 1 |  |
| Diversity of HT | - | - | ~ 1 | ~ 1 |  |


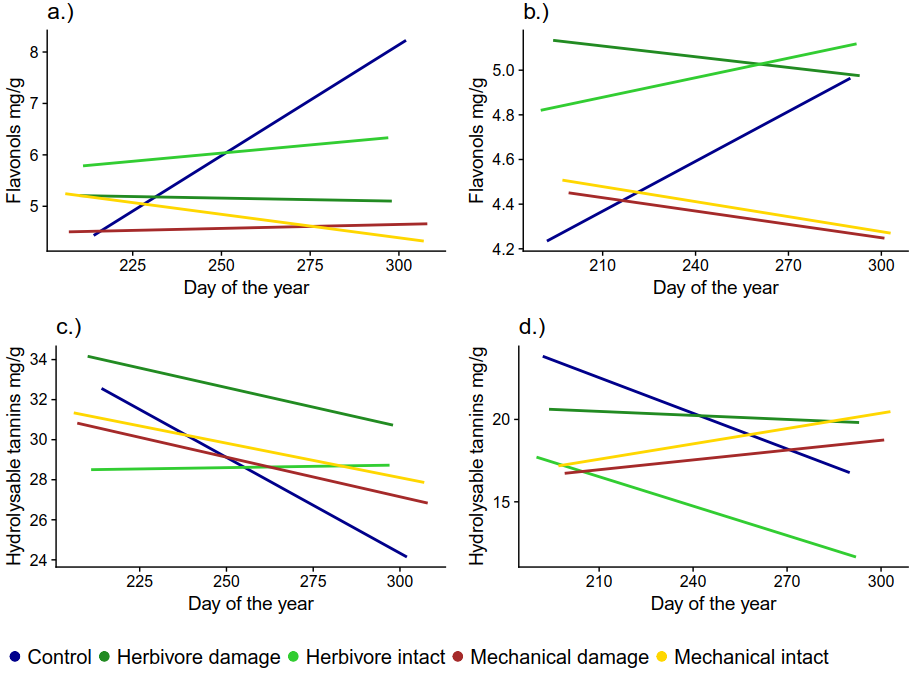


**Figure B1.** Predicted values for the response variable which showed significant site × treatment or date × treatment interactions over the sampling period. Panel a) shows flavonol concentration in Wytham Woods, b) flavonol concentration by the John Krebs field station, c) hydrolysable tannin concentration in Wytham Woods and d) hydrolysable tannin concentration by the John Krebs field station.

**File C. The literature review on the relationship between herbivory and polyphenol chemistry.**

For many of the compounds, we found only handful of studies reporting the relationship between the compound and herbivory. For these compounds, we report the results from all studies found in the literature search. For chlorogenic acid there were considerably more studies (n = 99). Thus, for this compound, we report only a few examples of the effects reported in the literature. For five compounds, we found no studies testing their relationship with herbivory. Thus, to our knowledge, for these compounds (casuarinin, galloyl-HHDP-glucose, stachyurin, quercetin malonylglucoside, kaempferol malonylglucoside) our study is the first to test the relationship between herbivory and their concentration.

To investigate whether the relationships between individual polyphenol compounds and herbivory reported in the literature reflect the anti-herbivore potential of the compounds, we calculated two metrics for each compound: oxidative activity and protein precipitation activity. Oxidative activity reflects the anti-herbivore potential of the compound if the herbivore has a basic gut (pH 9–12) and no efficient detoxification systems. Protein precipitation activity reflects anti-herbivore activity if the herbivore does not have a basic gut, and can precipitate dietary proteins ([12]). The activity measures were estimated based on equations from previous literature (for hydrolysable tannins: [12,13], for other compounds: J-P Salminen, unpublished data; see Table C2 in File C in S1 Appendix).

We related the oxidative and protein precipitation activity to the percentage of the studies showing evidence for anti-herbivore activity for each compound separately. We built separate models using either the estimated activity, or a ranking of the compounds (from most active to least active) as explanatory variables. The latter was done in order to include also those compounds for which it was not possible to estimate the actual value. A study was determined to show antiherbivore activity if the results showed a negative correlation with herbivore abundance, negative correlation or effect on herbivore performance, or an increase in concentration of the compound after herbivory. We excluded chlorogenic acid from the analysis because we did not survey through all the literature for this compound. We built four generalized linear models with the proportion of studies showing evidence for anti-herbivore activity (as two column matrix of “successes” and “failures”) as a function of the oxidative activity ranking (from 1 to 27), the protein precipitation activity ranking, or the estimated values for either activities with a binomial error distribution and logit link.

Compounds that ranked low on both activity measures had a smaller proportion of studies showing evidence for anti-herbivore activity, though these trends were not significant (oxidative activity: z = −1.52, p = 0.13; protein precipitation activity: z = −1.77, p = 0.07; Figure C1 in File C in S1 Appendix). Compounds that ranked higher on both activity measures included those that showed evidence for anti-herbivore activity, as well as those that did not. There were no trends with the percentage of evidence and the estimated activity values (oxidative activity: z = 0.81, p = 0.42; protein precipitation activity: z = 1.57, p = 0.12). The lack of clarity in these trends is most likely due to the low resolution of the data: most compounds had very few studies on them, which often included evidence both for and against that compound acting as an anti-herbivore defence. Since the impact of the compound as a defence against herbivores can depend on the species identity of the herbivore [14], or even on the pH of its gut [12], compounds functioning as defences against certain herbivores are likely to show negative results against others.

**Table C1.** Summary table of all the phenolic compounds and documented relationships with herbivory from the literature search. Note that “no correlation” refers to observational studies for which it was not possible to separate the direction of the interaction (i.e. whether herbivory has not affected plant chemistry or *vice versa*).

| **Compound** | **Herbivory related effects** | **References** |
| --- | --- | --- |
| **Hydrolysable tannins** | |  |
| Vescalagin | Increase with simulated herbivory (1). Feeding deterrent (2). Positive correlation with leafminer abundance (3). No change after psyllid feeding (4). | 1. Moctezuma et al. 2014  2. Roslin & Salminen 2008  3. Yarnes et al. 2008  4. Patton et al. 2018 |
| Cocciferin D_2_ | Positive correlation with leafminers (1). No correlation with herbivory (2) | 1. Yarnes et al. 2008  2. Moctezuma et al. 2014 |
| Vescavaloninic acid | No correlation with herbivory. | Moctezuma et al. 2014 |
| Castalagin | Negative correlation with leafminers (1). No correlation with herbivory (2). | 1. Yarnes et al. 2008  2. Moctezuma et al. 2014 |
| Pedunculagin | Negative correlation with leafminers (1). Increased metabolic cost for herbivores, but no effect on herbivore performance (2). Positive correlation with larval survival and growth (3). Marginally non-significant increase after herbivory (4). | 1. Yarnes et al. 2008  2. Barbehenn et al. 2009  3. Haviola et al. 2007  4. Ruuhola et al. 2013 |
| Castavaloninic acid | Negative correlation with leafminers. | Yarnes et al. 2008 |
| Casuarictin | Negative correlation with herbivore weight (1). Increase after herbivory (2, 3). | 1. Ruuhola et al. 2007  2. Ruuhola et al. 2013  3. Ossipov et al. 2014 |
| Monogalloylglucose | No correlation with herbivory (1). Both positive and negative correlation with herbivory, depending on the level of previous herbivory (2). No correlation with caterpillar growth (4). No change after herbivory (3, 5). | 1. Ruuhola et al. 2007  2. Lempa et al. 2004  3. Patton et al. 2018  4. Tikkanen & Julkunen-Tiitto 2003  5. Ruuhola et al. 2013 |
| HHDP-glucose | No correlation with herbivory (1). No change after psyllid feeding (2). | 1. Moctezuma et al. 2014  2. Patton et al. 2018 |
| Tellimagrandin I | No effect on herbivores (1). No change after psyllid feeding (2). Increase after herbivory (3, 4). | 1. Haviola et al. 2007  2. Patton et al. 2018  3. Ruuhola et al. 2013  4. Ossipov et al. 2014 |
| Casuarinin | No previous data |  |
| Galloyl-HHDP-glucose | No previous data |  |
| Stachyurin | No previous data |  |
| Tellimagrandin II | No change after psyllid feeding (1). Increase after herbivory (2). | 1. Patton et al. 2018  2. Ruuhola et al. 2013 |
| Tetragalloylglucose | Negative effect on herbivore encapsulation (1). Lowered leaf consumption of early instars, no effect on late instars, no effect on herbivore performance (2). No change after psyllid feeding (3). Increase after herbivory (4). | 1. Haviola et al. 2007  2. Salminen & Lempa 2002  3. Patton et al. 2018  4. Ruuhola et al. 2013 |
| Pentagalloylglucose | Positive correlation with larval weight (1). Lowered leaf consumption of early instars, no effect on late instars, no effect on herbivore performance (2). Increase after psyllid feeding (3). | 1. Haviola et al. 2007  2. Salminen & Lempa 2002  3. Patton et al. 2018 |
| **Flavonols*** |  |  |
| Quercetin glucoside | No correlation with herbivory (1). Increase after psyllid feeding (2). No correlation with herbivore performance (4, 8). Not affected by oviposition or herbivory (5), by artificial defoliation (3), by bark beetle attack (6) or by MeJa -treatment (7). | 1. Moctezuma et al. 2014  2. Patton et al. 2018  3. Keinänen et al. 1999  4. Tikkanen & Julkunen-Tiitto 2003  5. Beyaert et al. 2012  6. Schiebe et al. 2012  7. Ruiz-Garcia et al. 2013  8. Lehrman et al. 2012 |
| Quercetin glucuronide | Negative correlation with beetle feeding (1). No correlation with herbivory (2). No change after psyllid feeding (3), artificial defoliation (4) or MeJa -treatment (5). | 1. Johnson et al. 2009  2. Moctezuma et al. 2014  3. Patton et al. 2018  4. Keinänen et al. 1999  5. Ruiz-Garcia et al. 2013 |
| Kaempferol glucoside | No correlation with herbivory (1). Increased resistance to thrips (2). No effect on herbivores (3, 5). Not induced by spruce bark beetle attack (4). | 1. Moctezuma et al. 2014  2. Leiss et al. 2009a  3. Tikkanen & Julkunen-Tiitto 2003  4. Schiebe et al. 2012  5. Lehrman et al. 2012 |
| Quercetin diglycoside | No change after herbivory (1). No effect on beetle oviposition or survival (2). | 1. O'Neil et al. 2010  2. Torp et al. 2013 |
| Kaempferol glucuronide | No correlation with herbivory (1). No effect of artificial defoliation (2). | 1. Johnson et al. 2009  2. Keinänen et al. 1999 |
| Quercetin malonylglucoside | No previous data |  |
| Kaempferol malonylglucoside | No previous data |  |
| Kaempferol diglycoside | Increase after beetle damage (1). No change after caterpillar damage (1). No effect on herbivores (2, 3). | 1. O'Neil et al. 2010  2. Torp et al. 2013  3. Lehrman et al. 2012 |
| **Other polyphenols** |  |  |
| PC dimer | Induced in phloem by simulated bark beetle attack (1). Decrease in phloem after mechanical wounding (2). | 1. Mason et al. 2017  2. Muilenburg et al. 2013 |
| Chlorogenic acid | Induced by herbivory (4, 11, 12), decrease after herbivory (2), no change after herbivory (9), no effect on herbivores (1, 7, 8, 13), negative effects on herbivores (1, 6), feeding deterrent (30), feeding stimulant (3, 10), increased resistance to herbivores (5). | 1. Pascacio-Villafán et al. 2014  2. Kirakosyan et al. 2004  3. Fulcher et al. 1998  4. Poveda et al. 2012  5. Leiss et al. 2009b  6. Felton et al. 1992  7. Ruuhola et al. 2007  8. Torp et al. 2013  9. Patton et al. 2018  10. Lempa et al. 2004  11. Zhang et al. 2017  12. Kessler & Baldwin 2004  13. Lehrman et al. 2012 |
| Catechin | Induced by herbivory (3, 6, 7, 8, 10, 14), decrease after herbivory or simulated herbivory (1, 9, 11, 17, 19), not affected by herbivory (15, 16), negative effects on herbivores (2, 5), positive effects on herbivores (5, 18), no effect on herbivores (13), feeding deterrent (10, 12), positive correlation with natural herbivory (1), increased resistance to herbivores (4). | 1. Moctezuma et al. 2014  2. Ruuhola et al. 2007  3. Zhang et al. 2017  4. Duan et al. 2014  5. Pascacio-Villafán et al. 2014  6. Usha Rani et al. 2013  7. Roitto et al. 2009  8. Thelen et al. 2005  9. Kirakosyan et al. 2004  10. Berg 2003  11. Keinänen et al. 1999  12. Fulcher et al. 1998  13. Tikkanen & and Julkunen-Tiitto 2003  14. Ossipov et al. 2015  15. Beyaert et al. 2012  16. Schiebe et al. 2012  17. Ruiz-Garcia et al. 2013  18. Lehrman et al. 2012  19. Muilenburg et al. 2013 |

*** These names refer to groups of compounds, not individual chemicals. Thus, studies referring to this compound group might have examined different individual compounds.**

*References for Table C1*

Barbehenn RV, Jaros A, Lee G, Mozola C, Weir Q, Salminen J-P. Tree resistance to Lymantria dispar caterpillars: importance and limitations of foliar tannin composition. Oecologia. 2009 Apr;159(4):777–88.

Berg TB. Catechin content and consumption ratio of the collared lemming. Oecologia. 2003 Apr;135(2):242–9.

Beyaert I, Kopke D, Stiller J, Hammerbacher A, Yoneya K, Schmidt A, et al. Can insect egg deposition ‘warn’ a plant of future feeding damage by herbivorous larvae? Proceedings of the Royal Society B: Biological Sciences. 2012 Jan 7;279(1726):101–8.

Duan C-X, Zhu Z-D, Ren G-X, Wang X-M, Li D-D. Resistance of Faba bean and pea germplasm to Callosobruchus chinensis (Coleoptera: Bruchidae) and its relationship with quality components. Journal of Economic Entomology. 2014 Oct 1;107(5):1992–9.

Felton GW, Donato KK, Broadway RM, Duffey SS. Impact of oxidized plant phenolics on the nutritional quality of dietar protein to a noctuid herbivore, Spodoptera exigua. Journal of Insect Physiology. 1992 Apr;38(4):277–85.

Fulcher AF, Ranney TG, Burton JD, Walgenbach JF, Danehower, D. A. Role of foliar phenolics in host plant resistance of Malus taxa to adult Japanese beetles. Hortscience. 1998;33(5):862–5.

Haviola S, Kapari L, Ossipov V, Rantala MJ, Ruuhola T, Haukioja E. Foliar phenolics are differently associated with Epirrita autumnata growth and immunocompetence. Journal of Chemical Ecology. 2007 Apr 26;33(5):1013–23.

Johnson MTJ, Agrawal AA, Maron JL, Salminen J-P. Heritability, covariation and natural selection on 24 traits of common evening primrose ( *Oenothera biennis* ) from a field experiment. Journal of Evolutionary Biology. 2009 Jun;22(6):1295–307.

Keinänen M, Julkunen-Tiitto R, Mutikainen P, Walls M, Ovaska J, Vapaavuori E. Trade-offs in phenolic metabolism of Silver birch: effects of fertilization, defoliation, and genotype. Ecology. 1999 Sep;80(6):1970–86.

Kessler A, Baldwin IT. Herbivore-induced plant vaccination. Part I. The orchestration of plant defenses in nature and their fitness consequences in the wild tobacco *Nicotiana attenuata*. The Plant Journal. 2004 May;38(4):639–49.

Kirakosyan A, Kaufman P, Warber S, Zick S, Aaronson K, Bolling S, et al. Applied environmental stresses to enhance the levels of polyphenolics in leaves of hawthorn plants. Physiologia Plantarum. 2004 Jun;121(2):182–6.

Lehrman A, Torp M, Stenberg JA, Julkunen-Tiitto R, Björkman C. Estimating direct resistance in willows against a major insect pest, Phratora vulgatissima, by comparing life history traits: Estimating direct resistance against Phratora vulgatissima in willows. Entomologia Experimentalis et Applicata. 2012 Jul;144(1):93–100.

Leiss KA, Maltese F, Choi YH, Verpoorte R, Klinkhamer PGL. Identification of chlorogenic acid as a resistance factor for thrips in Chrysanthemum. Plant Physiology. 2009 Jul 1;150(3):1567–75.

Leiss KA, Choi YH, Abdel-Farid IB, Verpoorte R, Klinkhamer PGL. NMR metabolomics of thrips (Frankliniella occidentalis) resistance in Senecio hybrids. Journal of Chemical Ecology. 2009 Feb;35(2):219–29.

Lempa K, Agrawal AA, Salminen J-P, Turunen T, Ossipov V, Ossipova S, et al. Rapid herbivore-induced changes in mountain birch phenolics and nutritive compounds and their effects on performance of the major defoliator, Epirrita autumnata. Journal of Chemical Ecology. 2004 Feb;30(2):303–21.

Mason CJ, Villari C, Keefover-Ring K, Jagemann S, Zhu J, Bonello P, et al. Spatial and temporal components of induced plant responses in the context of herbivore life history and impact on host. Rasmann S, editor. Functional Ecology. 2017 Nov;31(11):2034–50.

Moctezuma C, Hammerbacher A, Heil M, Gershenzon J, Méndez-Alonzo R, Oyama K. Specific polyphenols and tannins are associated with defense against insect herbivores in the tropical oak Quercus oleoides. Journal of Chemical Ecology. 2014 May;40(5):458–67.

Muilenburg VL, Phelan PL, Bonello P, Loess PF, Herms DA. Characterization of wound responses of stems of paper birch (Betula papyrifera) and European white birch (Betula pendula). Trees. 2013 Aug;27(4):851–63.

O’Neill BF, Zangerl AR, Dermody O, Bilgin DD, Casteel CL, Zavala JA, et al. Impact of elevated levels of atmospheric CO2 and herbivory on flavonoids of soybean (Glycine max Linnaeus). Journal of Chemical Ecology. 2010 Jan;36(1):35–45.

Ossipov V, Klemola T, Ruohomäki K, Salminen J-P. Effects of three years’ increase in density of the geometrid Epirrita autumnata on the change in metabolome of mountain birch trees (Betula pubescens ssp. czerepanovii). Chemoecology. 2014 Oct;24(5):201–14.

Pascacio-Villafán C, Lapointe S, Williams T, Sivinski J, Niedz R, Aluja M. Mixture-amount design and response surface modeling to assess the effects of flavonoids and phenolic acids on developmental performance of Anastrepha ludens. Journal of Chemical Ecology. 2014 Mar;40(3):297–306.

Patton MF, Arena GD, Salminen J-P, Steinbauer MJ, Casteel CL. Transcriptome and defence response in *Eucalyptus camaldulensis* leaves to feeding by *Glycaspis brimblecombei* Moore (Hemiptera: Aphalaridae): a stealthy psyllid does not go unnoticed: *Eucalyptus-Glycaspis* psyllid interactions. Austral Entomology. 2018 May;57(2):247–54.

Poveda K, Gómez Jiménez MI, Halitschke R, Kessler A. Overcompensating plants: their expression of resistance traits and effects on herbivore preference and performance. Entomologia Experimentalis et Applicata. 2012 Jun;143(3):245–53.

Roitto M, Rautio P, Markkola A, Julkunen-tiitto R, Varama M, Saravesi K, et al. Induced accumulation of phenolics and sawfly performance in Scots pine in response to previous defoliation. Tree Physiology. 2008 Dec 5;29(2):207–16.

Roslin T, Salminen J-P. Specialization pays off: contrasting effects of two types of tannins on oak specialist and generalist moth species. Oikos. 2008 Oct;117(10):1560–8.

Ruiz-García Y, Gil-Muñoz R, López-Roca JM, Martínez-Cutillas A, Romero-Cascales I, Gómez-Plaza E. Increasing the phenolic compound content of grapes by preharvest application of abcisic acid and a combination of methyl jasmonate and benzothiadiazole. Journal of Agricultural and Food Chemistry. 2013 Apr 24;61(16):3978–83.

Ruuhola T, Salminen J-P, Haviola S, Yang S, Rantala MJ. Immunological memory of mountain birches: effects of phenolics on performance of the autumnal moth depend on herbivory history of trees. Journal of Chemical Ecology. 2007 May 25;33(6):1160–76.

Ruuhola T, Salminen P, Salminen J-P, Ossipov V. Ellagitannins: defences of *Betula nana* against *Epirrita autumnata* folivory? Agricultural and Forest Entomology. 2013 May;15(2):187–96.

Salminen J-P, Lempa K. Effects of hydrolysable tannins on a herbivorous insect: fate of individual tannins in insect digestive tract. Chemoecology. 2002 Nov;12(4):203–11.

Schiebe C, Hammerbacher A, Birgersson G, Witzell J, Brodelius PE, Gershenzon J, et al. Inducibility of chemical defenses in Norway spruce bark is correlated with unsuccessful mass attacks by the spruce bark beetle. Oecologia. 2012 Sep;170(1):183–98.

Thelen GC, Vivanco JM, Newingham B, Good W, Bais HP, Landres P, et al. Insect herbivory stimulates allelopathic exudation by an invasive plant and the suppression of natives: Allelopathic exudation by an invasive plant. Ecology Letters. 2005 Jan 13;8(2):209–17.

Tikkanen O-P, Julkunen-Tiitto R. Phenological variation as protection against defoliating insects: the case of Quercus robur and Operophtera brumata. Oecologia. 2003 Jul 1;136(2):244–51.

Torp M, Lehrman A, Stenberg JA, Julkunen-Tiitto R, Björkman C. Performance of an herbivorous leaf beetle (Phratora vulgatissima) on Salix F2 Hybrids: the importance of phenolics. Journal of Chemical Ecology. 2013 Apr;39(4):516–24.

Usha Rani P, Pratyusha S. Defensive role of Gossypium hirsutum L. anti-oxidative enzymes and phenolic acids in response to Spodoptera litura F. feeding. Journal of Asia-Pacific Entomology. 2013 Jun;16(2):131–6.

Yarnes CT, Boecklen WJ, Salminen J-P. No simple sum: seasonal variation in tannin phenotypes and leaf-miners in hybrid oaks. Chemoecology. 2008 Mar;18(1):39–51.

Zhang X, Sun X, Zhao H, Xue M, Wang D. Phenolic compounds induced by Bemisia tabaci and Trialeurodes vaporariorum in Nicotiana tabacum L. and their relationship with the salicylic acid signaling pathway. Arthropod-Plant Interactions. 2017 Oct;11(5):659–67.

**Table C2.** Summary table of the phenolic compounds investigated in the literature review. The table shows the percentage of published studies showing evidence for anti-herbivore activity, rankings of compounds based on their oxidative activity and protein precipitation activity (“1” denotes the highest activity in relation to the other compounds) and the calculated values for both activity measures (based on equations in [12,13]). Oxidative activity is calculated as the maximum rate of oxidation (mAbs / s / mM), and precipitation activity as the average insoluble complex formation (Abs) for the 0.1-1.0 mM concentration range. For explanations for these two units, see [12,13].

| **Compound** | **% of studies showing evidence for antiherbivore activity** | **Number of studies** | | **Oxidative activity ranking** | | **Protein precipitation activity ranking** | | **Oxidative activity (mAbs / s / mM)** | | **Protein precipitation activity (Abs)** |
| --- | --- | --- | --- | --- | --- | --- | --- | --- | --- | --- |
| **Hydrolysable tannins** |  |  |  | |  | |  | |  | |
| Vescalagin | 50 | 4 | 5 | | 12 | | 10.3 | | 0.3 | |
| Cocciferin D_2_ | 0 | 2 | 4 | | 1 | | 10.1 | | 1.8 | |
| Vescavaloninic acid | 0 | 1 | 2 | | 10 | | 21 | | 0.5 | |
| Castalagin | 50 | 2 | 3 | | 7 | | 13 | | 0.7 | |
| Pedunculagin | 50 | 4 | 8 | | 15 | | 4.8 | | 0.1 | |
| Castavaloninic acid | 100 | 1 | 1 | | 8 | | 23.3 | | 0.9 | |
| Casuarictin | 100 | 2 | 11 | | 5 | | 4 | | 0.8 | |
| Monogalloylglucose | 20 | 5 | 15 | | 16 | | 1 | | 0 | |
| HHDP-glucose | 0 | 2 | 9 | | 17 | | 3.9 | | 0 | |
| Tellimagrandin I | 50 | 4 | 12 | | 13 | | 2.4 | | 0.2 | |
| Casuarinin | NA | 0 | 6 | | 9 | | 7.9 | | 0.7 | |
| Galloyl-HHDP-glucose | NA | 0 | 10 | | 14 | | 3 | | 0.2 | |
| Stachyurin | NA | 0 | 7 | | 11 | | 5.2 | | 0.3 | |
| Tellimagrandin II | 50 | 2 | 13 | | 3 | | 2 | | 1 | |
| Tetragalloylglucose | 75 | 4 | 16 | | 4 | | 0.7 | | 0.8 | |
| Pentagalloylglucose | 66 | 3 | 17 | | 2 | | 0.6 | | 1.2 | |
| **Flavonols** |  |  |  | |  | |  | |  | |
| Quercetin glucoside | 13 | 8 | 21 | | 21 | | NA | | 0 | |
| Quercetin glucuronide | 20 | 5 | 22 | | 20 | | NA | | 0 | |
| Kaempferol glucoside | 20 | 5 | 25 | | 26 | | 0 | | 0 | |
| Quercetin diglycoside | 0 | 2 | 20 | | 22 | | NA | | 0 | |
| Kaempferol glucuronide | 0 | 2 | 26 | | 25 | | 0 | | 0 | |
| Quercetin malonylglucoside | NA | 0 | 23 | | 19 | | NA | | 0 | |
| Kaempferol malonylglucoside | NA | 0 | 27 | | 24 | | 0 | | 0 | |
| Kaempferol diglycoside | 25 | 4 | 24 | | 27 | | 0 | | 0 | |
| **Other polyphenols** |  |  |  | |  | |  | |  | |
| PC dimer | 50 | 2 | 19 | | 6 | | NA | | NA | |
| Chlorogenic acid | Not analyzed |  | 14 | | 23 | | NA | | 0 | |
| Catechin | 58 | 19 | 18 | | 18 | | NA | | NA | |


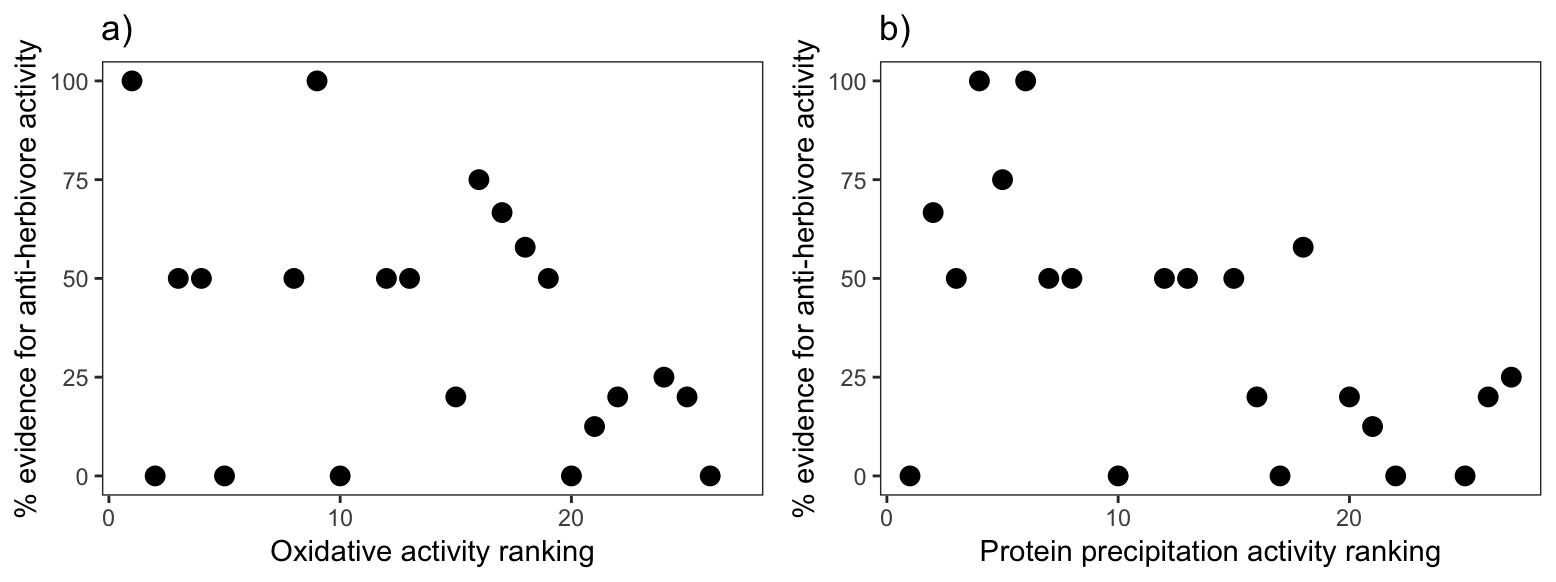


**Figure C1**. The relationship between the percentage of studies showing evidence for anti-herbivore activity and the ranking of compounds in terms of a) oxidative activity and b) protein precipitation activity. Each data point is a phenolic compound (Table C2 in File C in S1 Appendix), ranked from 1 to 27, with lower numbers denoting higher activity.

**Table A.** Summary of the investigated polyphenol compounds across different treatments.

**Table A.** Summary of the investigated polyphenol compounds and their mean concentrations (mg/g dry leaf mass) overall and across different experimental treatments. Compounds are listed from highest to lowest mean concentration. HT = hydrolysable tannin, PR= proanthocyanidin, FL = flavonol, PA =phenolic acid.

| **Compound** | **Type** | **Mean** | **Control** | **Herbivore damage** | **Herbivore intact** | **Mechanical damage** | **Mechanical intact** |
| --- | --- | --- | --- | --- | --- | --- | --- |
| Cocciferin D_2_ | HT | 9.22 | 9.09 ± 1.28 | 10.1 ± 1.34 | 8.36 ± 1.65 | 8.84 ± 1.28 | 9.60 ± 1.58 |
| Vescalagin | HT | 7.92 | 7.92 ± 0.98 | 8.38 ± 0.73 | 7.10 ± 0.83 | 7.77 ± 0.68 | 8.28 ± 0.83 |
| Catechin | PR | 3.4 | 3.68 ± 0.40 | 3.07 ± 0.36 | 3.52 ± 0.43 | 3.37 ± 0.29 | 3.40 ± 0.34 |
| Vescavaloninic acid | HT | 2.82 | 2.78 ± 0.47 | 3.01 ± 0.48 | 2.62 ± 0.52 | 2.83 ± 0.43 | 2.83 ± 0.53 |
| Castalagin | HT | 1.63 | 1.67 ± 0.20 | 1.72 ± 0.14 | 1.49 ± 0.17 | 1.58 ± 0.15 | 1.64 ± 0.18 |
| Quercetin glucoside | FL | 1.53 | 1.44 ± 0.13 | 1.47 ± 0.16 | 1.63 ± 0.18 | 1.37 ± 0.14 | 1.80 ± 0.31 |
| Quercetin glucuronide | FL | 1.42 | 1.40 ± 0.13 | 1.50 ± 0.19 | 1.62 ± 0.21 | 1.20 ± 0.11 | 1.41 ± 0.17 |
| Kaempferol glucoside | FL | 1.2 | 1.25 ± 0.26 | 1.25 ± 0.27 | 1.47 ± 0.25 | 0.92 ± 0.16 | 1.17 ± 0.26 |
| Pedunculagin | HT | 1.12 | 1.06 ± 0.08 | 1.19 ± 0.08 | 1.05 ± 0.08 | 1.15 ± 0.07 | 1.14 ± 0.10 |
| Castavaloninic acid | HT | 1.09 | 1.07 ± 0.09 | 1.14 ± 0.08 | 1.00 ± 0.11 | 1.14 ± 0.10 | 1.10 ± 0.13 |
| PC dimer | PR | 0.81 | 0.95 ± 0.13 | 0.70 ± 0.09 | 0.89 ± 0.14 | 0.77 ± 0.09 | 0.79 ± 0.11 |
| Quercetin diglycoside | FL | 0.74 | 0.74 ± 0.09 | 0.74 ± 0.10 | 0.80 ± 0.12 | 0.73 ± 0.08 | 0.70 ± 0.10 |
| Chlorogenic acid | PA | 0.22 | 0.21 ± 0.07 | 0.26 ± 0.11 | 0.29 ± 0.13 | 0.17 ± 0.06 | 0.21 ± 0.07 |
| Casuarictin | HT | 0.17 | 0.16 ± 0.03 | 0.15 ± 0.04 | 0.28 ± 0.17 | 0.13 ± 0.03 | 0.13 ± 0.02 |
| HHDP-glucose | HT | 0.14 | 0.14 ± 0.01 | 0.14 ± 0.008 | 0.13 ± 0.01 | 0.14 ± 0.008 | 0.13 ± 0.01 |
| Monogalloylglucose | HT | 0.13 | 0.14 ± 0.02 | 0.11 ± 0.02 | 0.15 ± 0.04 | 0.12 ± 0.02 | 0.13 ± 0.02 |
| Kaempferol glucuronide | FL | 0.12 | 0.12 ± 0.01 | 0.13+ ± 0.02 | 0.14 ± 0.02 | 0.10 ± 0.01 | 0.11 ± 0.02 |
| Quercetin malonylglucoside | FL | 0.08 | 0.07 ± 0.01 | 0.08 ± 0.02 | 0.09 ± 0.03 | 0.07 ± 0.02 | 0.08 ± 0.02 |
| Tellimagrandin I | HT | 0.07 | 0.08 ± 0.01 | 0.07 ± 0.01 | 0.06 ± 0.01 | 0.07 ± 0.01 | 0.07 ± 0.01 |
| Kaempferol malonylglucoside | FL | 0.04 | 0.04±0.01 | 0.05 ± 0.01 | 0.05 ± 0.01 | 0.04 ± 0.008 | 0.04 ± 0.009 |
| Galloyl-HHDP-glucose | HT | 0.03 | 0.03 ± 0.003 | 0.03 ± 0.002 | 0.03 ± 0.003 | 0.03 ± 0.003 | 0.03 ± 0.003 |
| Casuarinin | HT | 0.03 | 0.02 ± 0.002 | 0.04 ± 0.02 | 0.05 ± 0.03 | 0.02 ± 0.003 | 0.02 ± 0.002 |
| Kaempferol diglycoside | FL | 0.03 | 0.03 ± 0.01 | 0.04 ± 0.01 | 0.04 ± 0.01 | 0.01 ± 0.003 | 0.02 ± 0.01 |
| Tellimagrandin II | HT | 0.02 | 0.02 ± 0.004 | 0.02 ± 0.01 | 0.02 ± 0.008 | 0.02 ± 0.004 | 0.02 ± 0.01 |
| Stachyurin | HT | 0.01 | 0.01 ± 0.001 | 0.02 ± 0.01 | 0.02 ± 0.01 | 0.01 ± 0.001 | 0.01 ± 0.001 |
| Tetragalloylglucose | HT | 0.001 | 0.0004 ± 0.0004 | 0.001 ± 0.001 | 0.002 ± 0.002 | 0.0004 ± 0.0004 | 0.0004 ± 0.0004 |
| Pentagalloylglucose | HT | 0.0003 | 0 | 0.0004 ± 0.004 | 0.001 ± 0.001 | 0 | 0 |

**Table B.** Summary table for the statistical models.

**Table B.** Summary of statistical models assessing the effect of treatment on the studied leaf traits (mixed effect models), and the relationship between photosynthesis and leaf nitrogen content (linear models). Shown are coefficient estimates for fixed effects and the explanatory variables included in the final model (i.e. variables remaining after model selection; see methods). For each model, the intercept indicates the mean value of the response variable for a given level of the fixed effect(s) and for a given covariate value (0); as stated in parentheses. The other estimates indicate the mean change from the intercept, caused by the other fixed factor levels and by a unit change in covariate value. Date was expressed as Julian day, so that day = 1 is January 1^st^.

| **Response** | **Final model** | **Effects** | **Estimate** | **STD error** | **t value** |
| --- | --- | --- | --- | --- | --- |
| **The effect of treatment (mixed effect models)** | | | | | |
| LMA | ~ Proportion of damage + Dry mass | Intercept | 52.0 | 2.99 | 17.4 |
|  |  | Proportion of damage | 14.0 | 6.19 | 2.25 |
|  |  | Leaf mass | 0.06 | 0.008 | 8.15 |
| Leaf area | ~ 1 | Intercept | 19.5 | 2.63 | 7.40 |
| C:N | ~ Leaf mass | Intercept (mass = 0) | 1.36 | 0.017 | 80.6 |
|  |  | Leaf mass | −0.19 | 0.04 | −4.48 |
| C | ~ Leaf mass | Intercept | 46.5 | 0.8 | 61.6 |
|  |  | Leaf mass | −0.23 | 0.05 | −4.08 |
| N | ~ Site + Leaf mass | Intercept (Site=JK, mass=0) | 2.02 | 0.09 | 21.4 |
|  |  | Site WW | 0.23 | 0.11 | 2.03 |
|  |  | Leaf mass | 0.61 | 0.19 | 3.18 |
| Total polyphenols | ~ Date × Treatment | Intercept | 72.6 | 36.4 | 1.99 |
|  |  | Date | −0.20 | 0.16 | −1.20 |
|  |  | Herbivore damage | −40.1 | 49.3 | −0.82 |
|  |  | Herbivore intact | −9.02 | 51.5 | −0.18 |
|  |  | Mechanical damage | 96.0 | 52.1 | 1.84 |
|  |  | Mechanical intact | 5.94 | 52.3 | 0.11 |
|  |  | Site WW | 9.68 | 4.98 | 1.94 |
|  |  | Date × Herb dmg | 0.19 | 0.22 | 0.85 |
|  |  | Date × Herb int | 0.04 | 0.23 | 0.15 |
|  |  | Date × Mec dmg | −0.45 | 0.24 | −1.90 |
|  |  | Date × Mec int | −0.03 | 0.24 | −0.13 |
| Total flavonols | ~ Date + Treatment + Site + Date × Treatment + Site × Treatment | Intercept (Date = 0, Site=JK, Control) | -23.3 | 10.6 | -2.20 |
|  |  | Date | 0.12 | 0.05 | 2.62 |
|  |  | Herbivore damage | 29.2 | 14.6 | 2.00 |
|  |  | Herbivore intact | 23.74 | 15.1 | 1.57 |
|  |  | Mechanical damage | 27.0 | 14.5 | 1.86 |
|  |  | Mechanical intact | 35.6 | 14.5 | 2.45 |
|  |  | Site (WW) | 2.65 | 1.08 | 2.45 |
|  |  | Date × Herb dmg | −0.13 | 0.07 | −1.97 |
|  |  | Date × Herb int | −0.11 | 0.07 | −1.56 |
|  |  | Date × Mec dmg | −0.12 | 0.06 | −1.86 |
|  |  | Date × Mec int | −0.16 | 0.07 | −2.44 |
|  |  | Site × Herb dmg | −2.39 | 1.19 | −2.00 |
|  |  | Site × Herb int | −1.16 | 1.24 | −0.94 |
|  |  | Site × Mec dmg | −2.51 | 1.15 | −2.17 |
|  |  | Site × Mec int | −2.77 | 1.17 | −2.37 |
| Total hydrolysable tannins | ~ Date + Treatment + Site + Date × Treatment + Site × Treatment | Intercept (Site=JK, Date=0, Control) | 129.7 | 36.4 | 3.56 |
|  |  | Site WW | 1.97 | 7.19 | 0.27 |
|  |  | Date | −0.48 | 0.16 | −2.99 |
|  |  | Herbivore damage | −185.2 | 48.9 | −3.79 |
|  |  | Herbivore intact | −63.8 | 48.9 | −1.30 |
|  |  | Mechanical damage | 40.8 | 48.7 | 0.84 |
|  |  | Mechanical intact | −111.3 | 48.7 | −2.29 |
|  |  | Site × Herb dmg | 10.2 | 3.74 | 2.73 |
|  |  | Site × Herb int | 5.99 | 3.75 | 1.60 |
|  |  | Site × Mec dmg | 6.53 | 3.66 | 1.78 |
|  |  | Site × Mec int | 11.6 | 3.67 | 3.16 |
|  |  | Date × Herb dmg | 0.83 | 0.22 | 3.78 |
|  |  | Date × Herb int | 0.27 | 0.22 | 1.22 |
|  |  | Date × Mec dmg | −0.21 | 0.22 | −0.98 |
|  |  | Date × Mec int | 0.47 | 0.22 | 2.17 |
| Diversity of all polyphenols | ~ Date | Intercept (Date =0) | 1.43 | 0.20 | 7.03 |
|  |  | Date | 0.003 | 0.001 | 3.65 |
| Diversity of flavonols | ~ 1 | Intercept | 1.45 | 0.04 | 38.2 |
| Diversity of hydrolysable tannins | ~ Date × Treatment | Intercept (Date=0, Control) | 1.11 | 0.25 | 4.44 |
|  |  | Date | 0.002 | 0.001 | 1.82 |
|  |  | Herbivore damage | 0.08 | 0.33 | 0.24 |
|  |  | Herbivore intact | -0.30 | 0.33 | −0.90 |
|  |  | Mechanical damage | -0.38 | 0.34 | −1.14 |
|  |  | Mechanical intact | 0.04 | 0.34 | −1.14 |
|  |  | Date × Herb dmg | −0.0004 | 0.002 | −0.30 |
|  |  | Date × Herb int | 0.001 | 0.001 | 0.90 |
|  |  | Date × Mec dmg | 0.002 | 0.002 | 1.19 |
|  |  | Date × Mec int | −0.0002 | 0.002 | −0.11 |
| **The relationships between photosynthesis and other leaf traits (linear models)** | | | | | |
| A_1000_ | ~ site + N | Intercept (Site = JK) | −5.9 | 4.8 | -1.2 |
|  |  | Site WW | 1.3 | 1.3 | 1.0 |
|  |  | N | 6.6 | 2.9 | 2.9 |

**Abbreviations: Leaf dmg = proprotion of damage per leaf, JK = John Krebs field station, WW = Wytham Woods, Herb dmg = damaged leaf in the herbivory treatment, Herb int = intact leaf in the herbviory treatment, Mec dmg = mechanically damaged leaf, Mec int = intact leaf in the mechnical damage treatment.**

**Figure A.** Relationship between concentrations of individual polyphenol compounds and photosynthetic rate A_1000_.


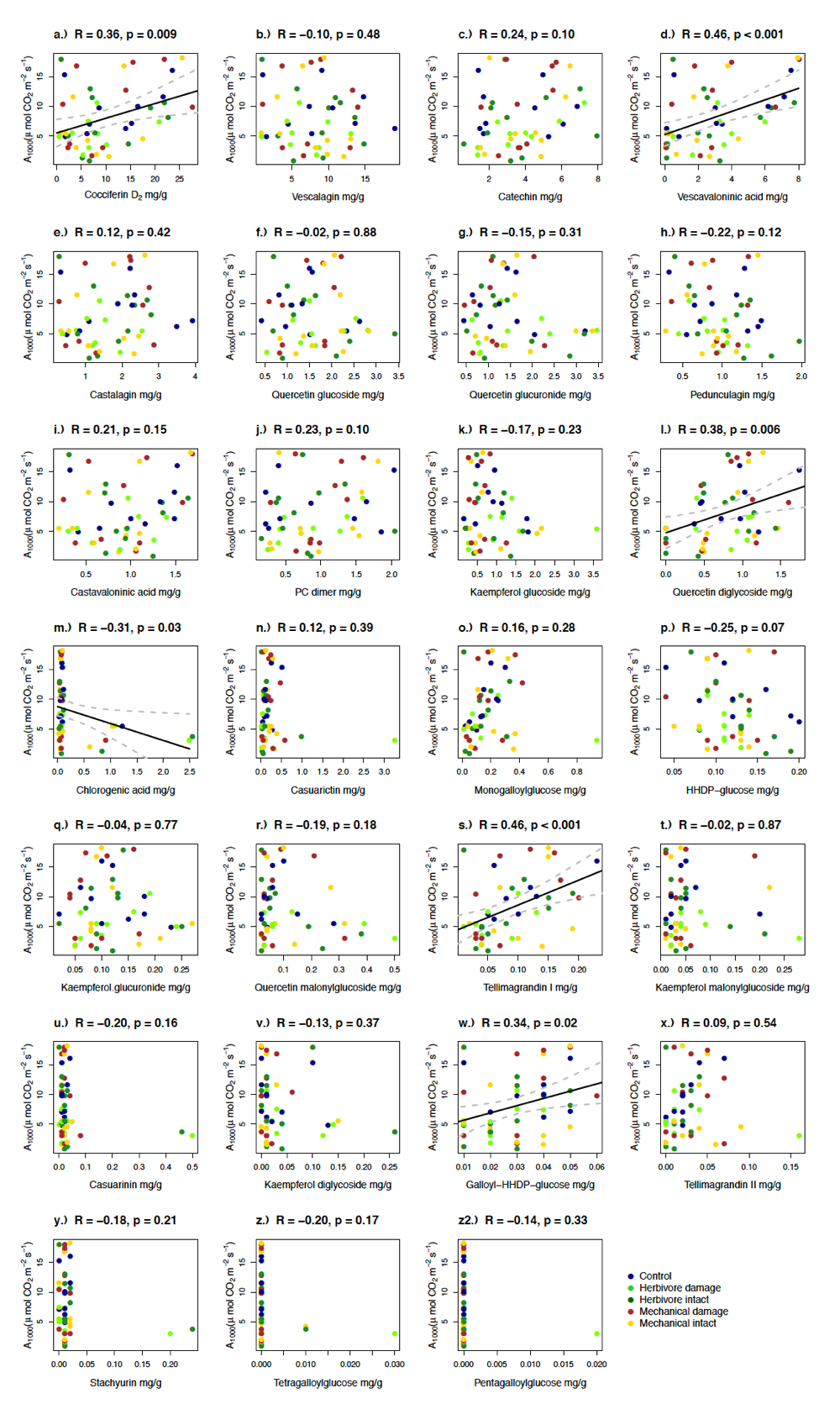


**S1 Figure.** Relationship between concentrations of individual polyphenol compounds and photosynthetic rate at 1000 µmol m^-2^ s^-1^ photosynthetically active radiation (parameter A_1000_). Pearson’s correlation coefficient (“R”) and the associated p-value are shown on each panel. For statistically significant relationships (p < 0.05), fitted values and 95% confidence intervals from a linear model describing the specific relationship are shown.

**Table C.** The unadjusted F and p-values per compound from the multivariate analysis.

**Table C.** The unadjusted F and p-values for each polyphenol compound separately from the multivariate analysis. Shown are the effects of the five experimental treatments (*herbivore damage, herbivore intact, mechanical damage, mechanical intact and control*), the two sites, the individual trees and the collection date. Statistically significant effects (p < 0.05) are highlighted in bold.

| **Compound** | **Site** |  | **Tree** |  | **Collection date** | | **Leaf treatment** | |  |
| --- | --- | --- | --- | --- | --- | --- | --- | --- | --- |
|  | F | p | F | p | F | p | F | P | |
| Cocciferin D_2_ | 35.5 | **0.002** | -11.8 | 0.38 | 248 | **0.002** | 2.34 | 0.06 | |
| Vescalagin | 7.90 | **0.01** | -11.8 | 0.46 | 245 | **0.002** | 2.23 | 0.08 | |
| Catechin | 3.97 | 0.06 | -11.7 | 0.76 | 241 | **0.002** | 2.13 | 0.09 | |
| Vescavaloninic acid | 95.6 | **0.002** | -11.0 | 0.30 | 244 | **0.002** | 2.36 | 0.06 | |
| Castalagin | 27.7 | **0.002** | -10.7 | 0.25 | 226 | **0.002** | 2.27 | 0.07 | |
| Quercetin glucoside | 3.14 | 0.08 | -8.90 | 0.45 | 166 | **0.002** | 1.67 | 0.17 | |
| Quercetin glucuronide | 1.81 | 0.20 | 0.35 | **0.02** | 64.2 | **0.049** | 1.18 | 0.32 | |
| Kaempferol glucoside | 8.72 | **0.008** | 3.27 | **0.002** | 6.41 | 0.30 | 0.83 | 0.48 | |
| Pedunculagin | 0.33 | 0.57 | 12.1 | **0.002** | 48.4 | **0.02** | 1.17 | 0.18 | |
| Castavaloninic acid | 24.2 | **0.002** | -3.77 | 0.06 | 128 | **0.002** | 2.25 | 0.06 | |
| PC-dimer | 0.95 | 0.32 | -6.35 | 0.12 | 108 | **0.003** | 1.31 | 0.19 | |
| Quercetin diglycoside | 25.1 | **0.002** | 25.3 | **0.002** | 82.1 | 0.05 | 1.14 | 0.32 | |
| Chlorogenic acid | 17.6 | **0.002** | -11.8 | 0.59 | 247 | **0.002** | 2.26 | 0.08 | |
| Casuarictin | 0.02 | 0.89 | -11.7 | 0.57 | 238 | **0.002** | 2.16 | 0.11 | |
| HHDP-glucose | 1.06 | 0.29 | -12.0 | 0.71 | 249 | **0.002** | 2.28 | 0.08 | |
| Monogalloylglucose | 29.5 | **0.002** | -11.8 | 0.57 | 241 | **0.002** | 2.21 | 0.09 | |
| Kaempferol glucuronide | 0.34 | 0.56 | -11.9 | 0.66 | 239 | **0.002** | 2.16 | 0.09 | |
| Quercetin malonylglucoside | 2.11 | 0.16 | -11.9 | 0.55 | 2489 | **0.002** | 2.24 | 0.08 | |
| Tellimagrandin I | 69.1 | **0.002** | -11.9 | 0.76 | 246 | **0.002** | 2.22 | 0.09 | |
| Kaempferol malonylglucoside | 8.64 | **0.005** | -11.9 | 0.60 | 252 | **0.002** | 2.29 | 0.08 | |
| Galloyl-HHDP-glucose | 84.2 | **0.002** | -12.0 | 0.83 | 248 | **0.002** | 2.27 | 0.08 | |
| Casuarinin | 3.01 | 0.10 | -12.0 | 0.78 | 248 | **0.002** | 2.25 | 0.08 | |
| Kaempferol diglycoside | 31.9 | **0.002** | -12.0 | 0.46 | 249 | **0.002** | 2.29 | 0.08 | |
| Tellimagrandin II | 11.4 | **0.003** | -11.9 | 0.62 | 249 | **0.002** | 2.28 | 0.08 | |
| Stachyurin | 0.02 | 0.91 | -12.0 | 0.80 | 249 | **0.002** | 2.28 | 0.08 | |
| Tetragalloylglucose | 1.99 | 0.21 | -12.0 | 0.81 | 250 | **0.002** | 2.29 | 0.07 | |
| Pentagalloylglucose | 0.14 | 0.69 | -12.0 | 0.78 | 250 | **0.002** | 2.29 | 0.07 | |

**Figure B.** Relationship between concentrations of individual polyphenol compounds and leaf respiration rate


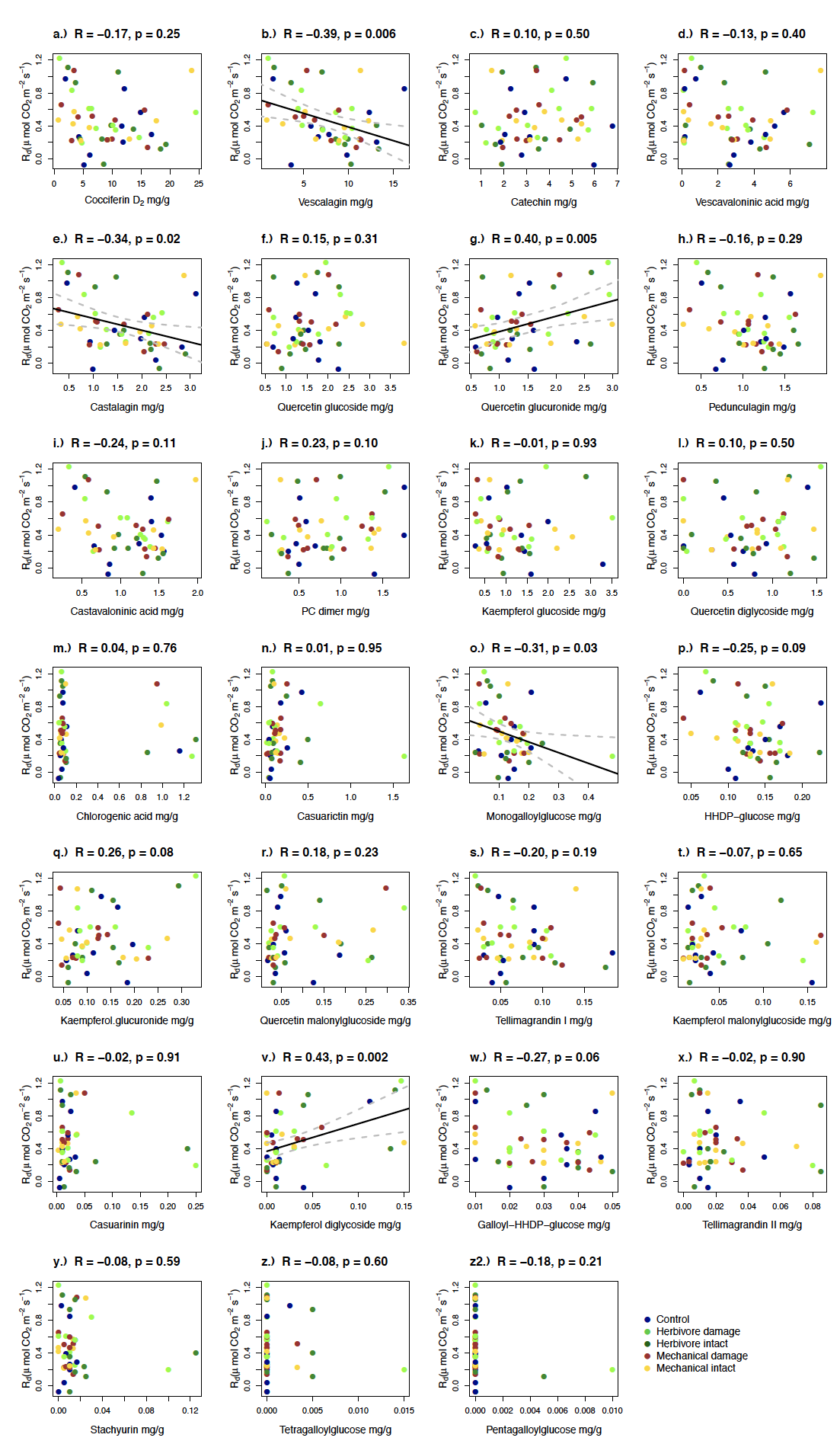


**Figure B.** Relationship between concentrations of individual polyphenol compounds and leaf respiration rate (parameter R_d_). Since leaf respiration and polyphenol concentrations were measured on different leaves and during different years, the data points represent averages over trees and leaf-level treatments per tree. Pearson’s correlation coefficient (“R”) and the p-value for the correlation are shown. For statistically significant relationships (p < 0.05), fitted values and 95% confidence intervals from a linear model describing the specific relationship are shown. Note that the respiration values on y-axis are shown as positive to make the graph more intuitive (i.e. so that larger respiration values correspond to higher respiration rate).

**Figure C.** Contributions of the different leaf traits to the PCA axes.


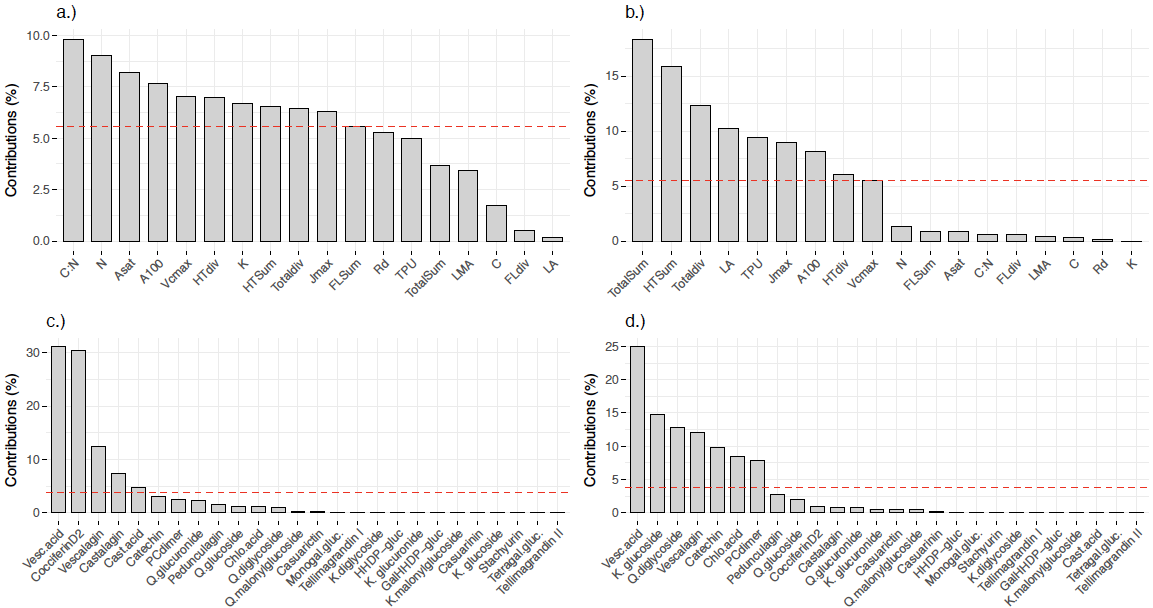


**Figure C.** Contributions of the different leaf traits to the PCA axes in Figure 4 in the main text. Panel a.) PC1 and b) PC2 for all traits except individual polyphenols. Panel c) PC1 and d) PC2 for the different polyphenol groups. The red reference line corresponds to the expected value if the contributions were uniform.

**Figure D.** Partial RDA analysis on all leaf traits except the individual polyphenol compounds.


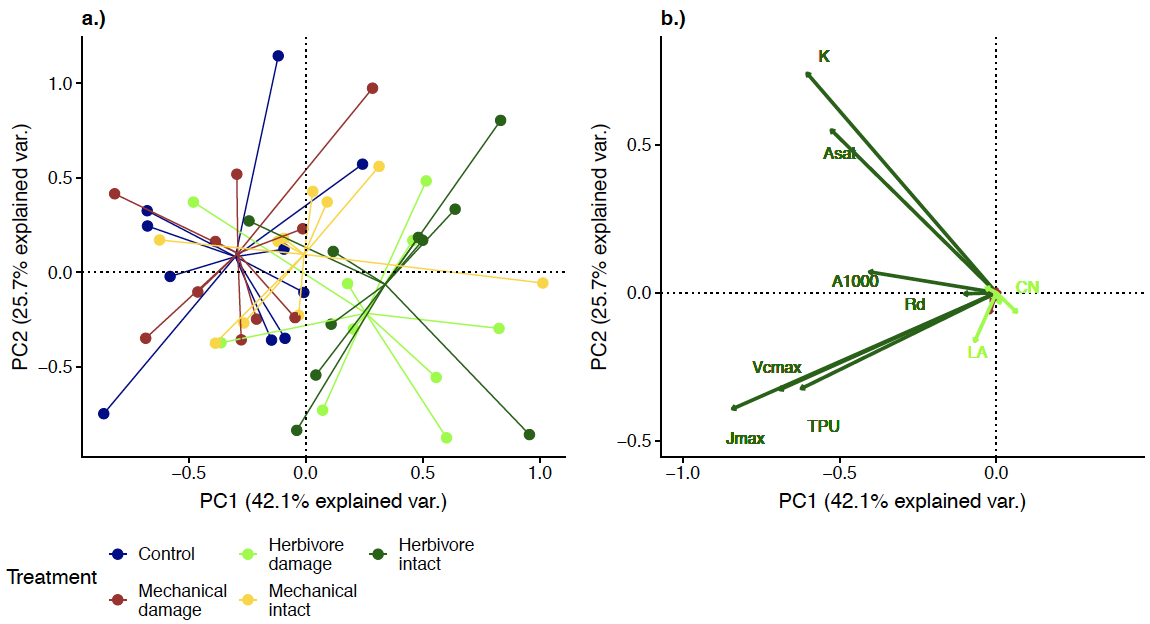


**Figure D.** Partial RDA analysis on all leaf traits except the individual polyphenol compounds, in which the variation caused by tree has been removed. Data are shown for the unconstrained analyses. Panel a) shows ordination with grouping by the experimental treatment, and panel b) shows the effect of different leaf traits to the ordination results. Data points represent averages over tree-treatment combinations and use imputed values for missing values of nitrogen and carbon content. In panel b) dark green arrows represent different photosynthetic parameters, brown arrows (visible only under the light green arrows) represent traits related to polyphenol chemistry, and light green arrows other traits.

**Figure E**. Relationships between polyphenol concentration and diversity, and between polyphenol concentration and leaf size.


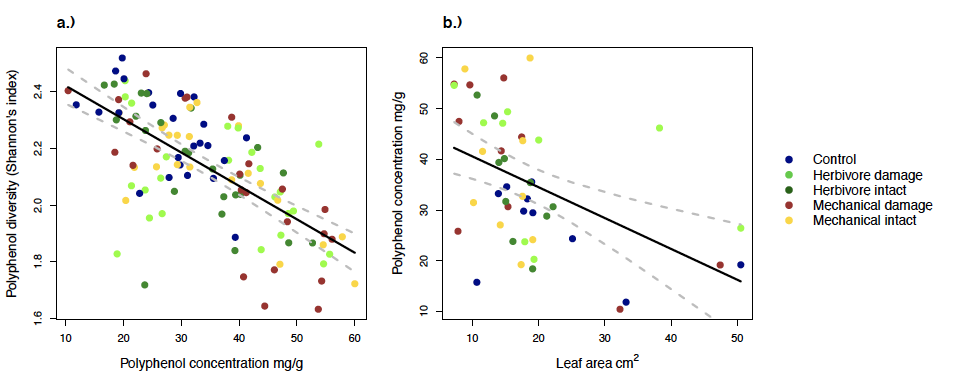


**Figure E.** Panel a) shows the relationship between concentration and diversity of polyphenols (Pearson's r = −0.67, p < 0.001). Panel b) shows the relationship between concentration of polyphenols and estimated leaf area without herbivory (Pearson's −0.47, p < 0.001).

**Figure F.** The effect of increasing sample size on the significance of the differences between different treatments.


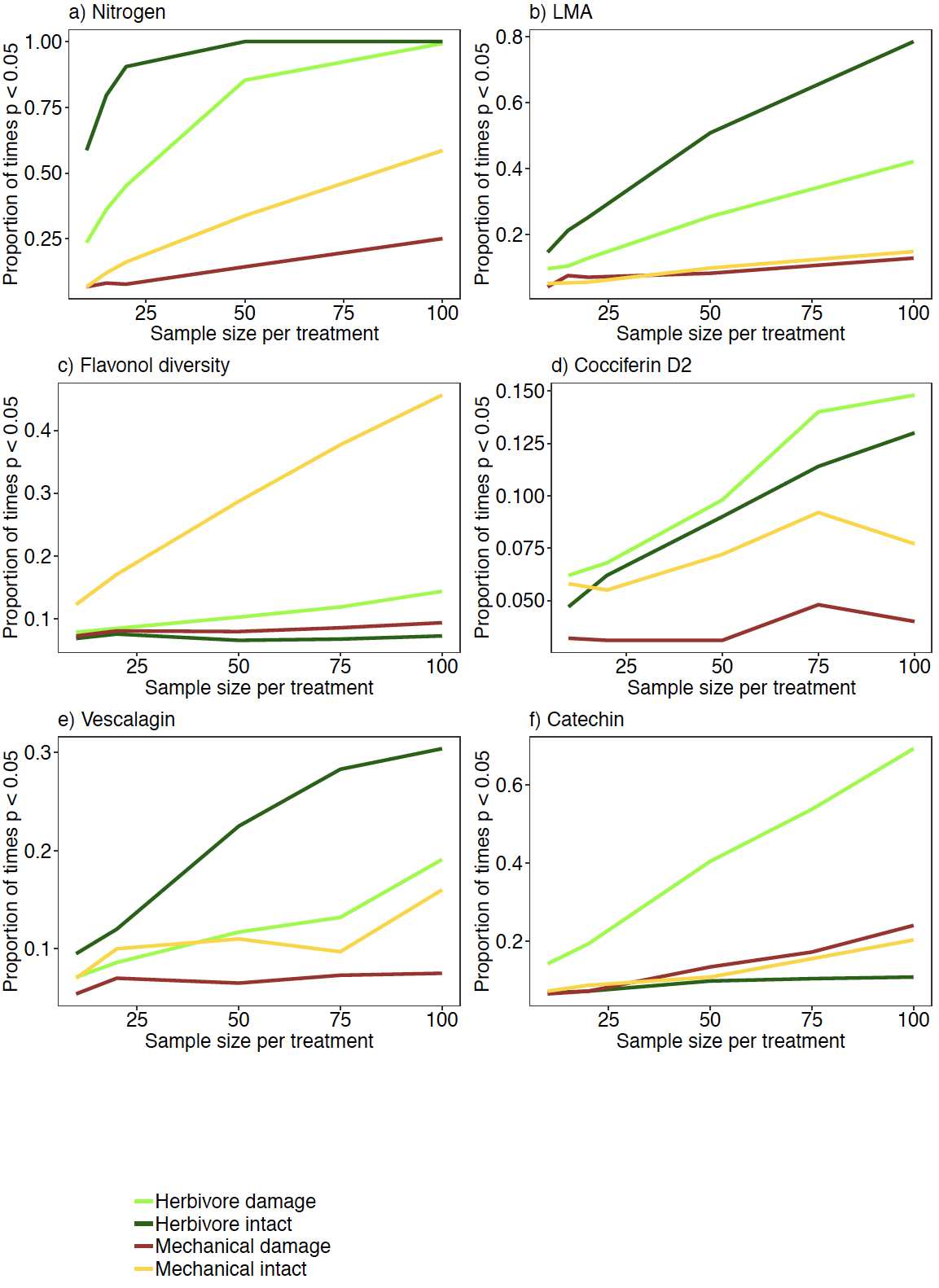


**Figure F.** The effect of increasing sample size on the significance of the differences in a) leaf nitrogen, b) LMA, c) flavonol diversity d) concentration of cocciferin D_2_, e) concentration of vescalagin and f) concentration of catechin between the four treatments when compared to the undamaged control leaf. The y-axis shows the proportion of times out of the 1000 simulations in which leaf treatment shows significant effect (p<0.05) in a linear model.

References for the Supplementary files

1. Visakorpi K, Gripenberg S, Malhi Y, Bolas C, Oliveras I, Harris N, et al. Small-scale indirect plant responses to insect herbivory could have major impacts on canopy photosynthesis and isoprene emission. New Phytologist. 2018;220. doi:10.1111/nph.15338

2. Morecroft MD, Stokes VJ, Morison JIL. Seasonal changes in the photosynthetic capacity of canopy oak (Quercus robur) leaves: the impact of slow development on annual carbon uptake. International Journal of Biometeorology. 2003;47: 221–226. doi:10.1007/s00484-003-0173-3

3. Volf M, Segar ST, Miller SE, Isua B, Sisol M, Aubona G, et al. Community structure of insect herbivores is driven by conservatism, escalation and divergence of defensive traits in *Ficus*. Ecology Letters. 2018;21: 83–92. doi:10.1111/ele.12875

4. Warton DI, Wright ST, Wang Y. Distance-based multivariate analyses confound location and dispersion effects: Mean-variance confounding in multivariate analysis. Methods in Ecology and Evolution. 2012;3: 89–101. doi:10.1111/j.2041-210X.2011.00127.x

5. Warton DI, Blanchet FG, O’Hara RB, Ovaskainen O, Taskinen S, Walker SC, et al. So many variables: joint modeling in community ecology. Trends in Ecology & Evolution. 2015;30: 766–779. doi:10.1016/j.tree.2015.09.007

6. Wang Y, Naumann U, Wright ST, Warton DI. mvabund - an R package for model-based analysis of multivariate abundance data: *The* mvabund R package. Methods in Ecology and Evolution. 2012;3: 471–474. doi:10.1111/j.2041-210X.2012.00190.x

7. Zuur AF, Ieno EN, Walker N, Saveliev AA, Smith GM. Mixed effects models and extensions in ecology with R. New York, NY, U.S.A: Springer; 2009.

8. Dray S, Josse J. Principal component analysis with missing values: a comparative survey of methods. Plant Ecology. 2015;216: 657–667. doi:10.1007/s11258-014-0406-z

9. Rubin DB. Inference and missing data. Biometrika. 1976;63: 581–592. doi:10.1093/biomet/63.3.581

10. Venables WN, Ripley BD. Modern applied statistics with S-PLUS. 3rd ed. New York: Springer; 1999.

11. Oksanen J. Multivariate analysis of ecological communities in R: vegan tutorial. 2015. Available: http://cc.oulu.fi/~jarioksa/opetus/metodi/vegantutor.pdf

12. Moilanen J, Salminen J-P. Ecologically neglected tannins and their biologically relevant activity: chemical structures of plant ellagitannins reveal their in vitro oxidative activity at high pH. Chemoecology. 2008;18: 73–83. doi:10.1007/s00049-007-0395-7

13. Engström MT, Arvola J, Nenonen S, Virtanen VTJ, Leppä MM, Tähtinen P, et al. Structural features of hydrolyzable tannins determine their ability to form insoluble complexes with bovine serum albumin. J Agric Food Chem. 2019;67: 6798–6808. doi:10.1021/acs.jafc.9b02188

14. Roslin T, Salminen J-P. Specialization pays off: contrasting effects of two types of tannins on oak specialist and generalist moth species. Oikos. 2008;117: 1560–1568. doi:10.1111/j.0030-1299.2008.16725.x
